# Integrative analysis of T cell motility from multi-channel microscopy data using TIAM

**DOI:** 10.1016/j.jim.2014.11.004

**Published:** 2015-01

**Authors:** Viveka Mayya, Willie Neiswanger, Ricardo Medina, Chris H. Wiggins, Michael L. Dustin

**Affiliations:** a Skirball Institute of Biomolecular Medicine, NYU Medical Center, 540 First Avenue, New York, NY 10016, USA; b Department of Applied Physics and Applied Mathematics, Columbia University, 200 S.W. Mudd Building, 500 W. 120th St., New York, NY 10027, USA; c Kennedy Institute of Rheumatology, University of Oxford, Roosevelt Drive, Headington, Oxford OX3 7FY, UK

**Keywords:** T cell motility, Tracking, Integrative analysis, Multi-channel microscopy

## Abstract

Integrative analytical approaches are needed to study and understand T cell motility as it is a highly coordinated and complex process. Several computational algorithms and tools are available to track motile cells in time-lapse microscopy images. In contrast, there has only been limited effort towards the development of tools that take advantage of multi-channel microscopy data and facilitate integrative analysis of cell-motility. We have implemented algorithms for detecting, tracking, and analyzing cell motility from multi-channel time-lapse microscopy data. We have integrated these into a MATLAB-based toolset we call TIAM (Tool for Integrative Analysis of Motility). The cells are detected by a hybrid approach involving edge detection and Hough transforms from transmitted light images. Cells are tracked using a modified nearest-neighbor association followed by an optimization routine to join shorter segments. Cell positions are used to perform local segmentation for extracting features from transmitted light, reflection and fluorescence channels and associating them with cells and cell-tracks to facilitate integrative analysis. We found that TIAM accurately captures the motility behavior of T cells and performed better than DYNAMIK, Icy, Imaris, and Volocity in detecting and tracking motile T cells. Extraction of cell-associated features from reflection and fluorescence channels was also accurate with less than 10% median error in measurements. Finally, we obtained novel insights into T cell motility that were critically dependent on the unique capabilities of TIAM. We found that 1) the CD45RO subset of human CD8 T cells moved faster and exhibited an increased propensity to attach to the substratum during CCL21-driven chemokinesis when compared to the CD45RA subset; and 2) attachment area and arrest coefficient during antigen-induced motility of the CD45A subset is correlated with surface density of integrin LFA1 at the contact.

## 1 Introduction

Mechanistic investigations into cell motility rely heavily on live-cell imaging and the subsequent analysis of time-lapse microscopy (TLM) data. A fundamental task herein is to perform automated tracking of cells. A variety of approaches have been developed for automated tracking of cells and also been made available to the research community as software packages or tools (Carpenter et al., 2006; de Chaumont et al., 2012; Meijering et al., 2012; Meijering et al., 2009; Padfield et al., 2011; Schindelin et al., 2012; Zimmer et al., 2006). In a common framework referred to as ‘tracking by detection’, cell detection is performed in each frame independently, and the detection results are joined together between frames via cell tracking algorithms. A popular basis for tracking known as the ‘nearest neighbor’ associates a detected cell in a given frame with the nearest detected cell in an adjacent frame. Recently, model-based methods have been developed for cell tracking (Dufour et al., 2011; Maska et al., 2014; Padfield et al., 2011). These methods comprise model-based representations of cells that evolve between subsequent frames to perform cell tracking.

Motility of cells is a highly complex, dynamic and coordinated mechano-chemical process that is influenced by hundreds of proteins (Lauffenburger and Horwitz, 1996; Parent and Weiner, 2013; Ridley et al., 2003). Study of T cell motility, along with that of other leukocytes, presents additional challenges when compared to the motility of cells of mesenchymal and epithelial origin. Leukocytes can move at speeds upwards of 10 μm/min and exhibit multiple modes of motility with remarkable flexibility to shift from one mode to the other (Friedl and Weigelin, 2008; Jacobelli et al., 2009; Lammermann and Sixt, 2009; Sixt, 2011). Leukocytes can also move with or without attachment to the substratum. Further, there is appreciable heterogeneity in the motility of leukocytes within a population. Thus, the study of leukocyte motility necessitates integrative experimental and analytical approaches to develop coherent understanding of the process (Zhang et al., 2013). Multi-channel or multi-mode microscopy offers a powerful platform to collect data and enable integrative analysis (Welch et al., 2011). An example of integrative analysis is relating polarization of a molecule of interest to thymocyte motility (Melichar et al., 2011; Pham et al., 2013). In order to conduct integrative analysis, one needs to be able to track cells and integrate information from multiple image series. Packages such as Volocity (from PerkinElmer), CellProfiler (Carpenter et al., 2006) and TACTICS (Pham et al., 2013) have the basic framework for tracking cells and associating information from additional image series to the tracks.

Interference reflection microscopy (IRM) provides information on adhesion and spreading on the substratum due to interference between light reflected from the cover-glass and the apposing cell membrane (Limozin and Sengupta, 2009). As T cells can move with or without attachment to the substratum and change contact area continuously, it is beneficial to include IRM along with fluorescence and transmitted light modes of microscopy. However, IRM is extremely sensitive to focus and planarity drifts as a result of which the IRM image series typically have spatiotemporally varying background and foreground intensity values. This presents a challenge to the aforementioned tools for integrative analysis as they rely on global thresholding for segmenting cells and generally report intensity values of additional channels upon global segmentation in the primary channel. It is desirable to treat individual image channels separately and also perform local segmentation.

In order to be able to accurately integrate IRM data, along with fluorescence and transmitted light data in 2D image series, we have developed a MATLAB-based toolset that we call ‘Tool for Integrative Analysis of Motility’ (TIAM). As a novel strategy, we have used centroid positions obtained from detection and tracking of cells to perform local segmentation for extracting features from transmitted light, reflection and fluorescence channels and then associating them back with cells and cell-tracks to facilitate integrative analysis. An intuitive user interface has been built onto TIAM to guide through the steps for choosing parameters to perform detection and subsequent analysis of motility characteristics. An additional user interface for dynamic visualization of selected tracks is also provided. As our main interest lies in T cell biology, we have validated the implemented algorithms on chemokine-induced and antigen-induced motility of human CD8 T cells and obtained novel insights that were critically dependent on the unique capabilities of TIAM.

## 2 Implementation

**Fig. 1:**
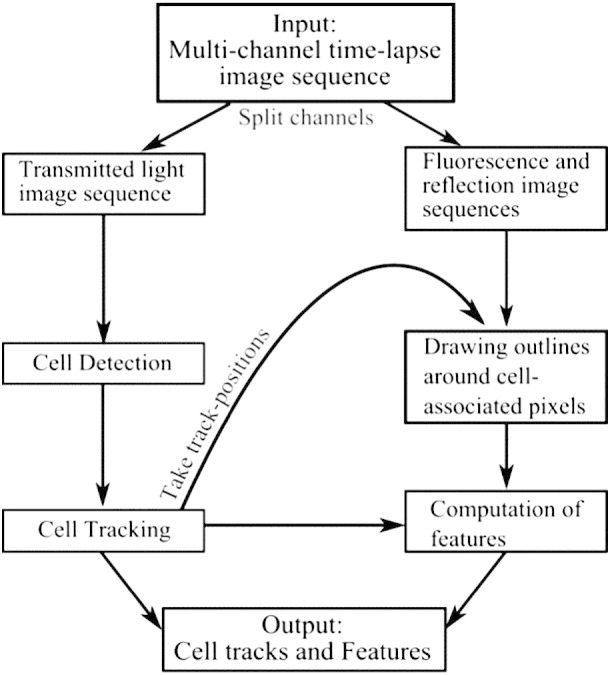
Overview of the schema for data integration in TIAM. Transmitted light images are used for detecting and tracking cells. Several parameters quantifying the motility characteristics are calculated and stored in MATLAB  ‘cell arrays’. Individual tracks are considered for extracting information from reflection and fluorescence images that are part of multi-channel time-lapse data. Centroids from track positions are used for local segmentation and outlining that would correspond to the cell under consideration. Features are computed from the outlined regions and stored along with rest of the track-related information.

The overall approach for integrative analysis of motility by TIAM is summarized in Fig. 1. Detection, tracking, feature extraction, and track editing algorithms were implemented in MATLAB (from MathWorks). The user interface to facilitate user-inputs was implemented in Java. A second user interface for dynamic visualization of individual or pairs of tracks was implemented in MATLAB. The TIAM software project has been deposited in GitHub for free access to the source code (https://github.com/willieneis/TIAM). A detailed user guide, demo and the URL link for benchmark datasets are provided in the Github repository. Additional description of algorithms can be found in the Supplementary methods section.

### 2.1 Detection of cells

TIAM is equipped to detect and track cells in transmitted light image series, such as those acquired by bright-field, differential interference contrast (DIC), or phase-contrast microscopy. We chose this approach for multiple reasons: a) Cell boundaries can be difficult to discern from fluorescence information when cells are in a crowded environment; the inherent nature of transmitted light imaging ensures that cell boundaries provide some contrast even in a crowded environment. b) Using transmitted light imaging for tracking of cells frees up a fluorescence channel for acquiring additional information about cells' behavior. c) Using transmitted light microscopy instead of fluorescence microscopy allows for long-term live-cell imaging as phototoxicity is minimized.

**Fig. 2:**
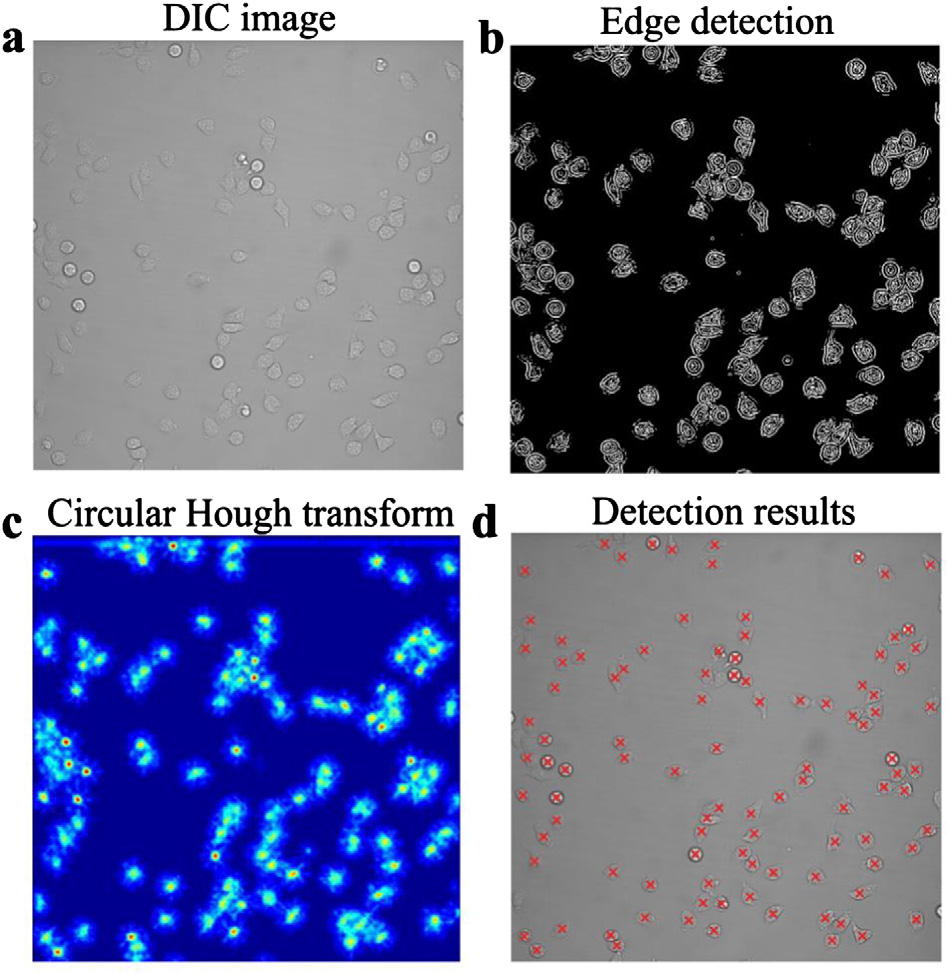
Detection and tracking of cells by TIAM. TIAM uses transmitted light images for detecting and tracking cells. Illustration of detection by TIAM is provided with an example (a–d). A DIC image of human primary CD8 T cells is used (a). The panels, b to d, represent sequential stages during cell detection. In the first step, the Canny edge filter is applied to generate a binary image of cell boundaries (b). Then, a circular Hough transform (CHT) is applied to this binary image. This operation maps cell outlines to points in a parameter space based on a voting scheme (c). Local maxima in the parameter space are used to pick centroids of cells (d). TIAM has a graphical user interface that walks the user through the choice of parameters for edge filtering and CHT to allow for accurate detection of cells.

TIAM's cell detection strategy involves finding cell-shaped patterns in the set of edges detected in an image. A Canny edge filter (Canny, 1986) is used to produce a binary image depicting all edges in a given video frame, and a circular Hough transform (CHT) (Duda and Hart, 1972) operates on this binary image to detect individual cells (Fig. 2a–d). This two-step strategy has been applied previously to detect nuclei in zebra fish embryos (Melani et al., 2007). The Hough transform is a robust method for detecting parameterized curves in images, where the task of detecting complex patterns of pixels (a costly global search problem) is transformed into the task of constructing peaks in a parameter space. The Hough transform carries out a voting process, where each edge pixel casts votes on curve parameters with which it is consistent; afterwards, the locations in the parameter space that have gained a sufficient number of votes are returned. Local maxima in this parameter space can be thought of as centroids of cells. This strategy is beneficial for detecting cells with low-contrast boundaries due to the ability of the CHT to detect shapes based on non-contiguous and partial set of edges. Furthermore, it bypasses the need for segmentation of individual cells and thus aid in the accuracy of detection in high-density environments (Fig. S1 for example). We have used Tao Peng's implementation of the CHT (CircularHough_Grd from the MATLAB File Exchange repository) as it considers a radius range during the voting process and includes an additional parameter for searching maxima over imperfect circular shapes. Accordingly, we have found our implementation to detect polarized T cells as well as cells of different types, morphologies and at different cellular densities in images acquired by all three aforementioned transmitted light microscopy techniques (Fig. 2, Fig. S1, Fig. S2, and Videos S1–3). The individual parameters involved in the detection step are described further in the Supplementary methods section. Parameter values typically used in our T cell imaging experiments are also provided.

Successful detection is critical for all the ensuing computational steps. Therefore we have developed a graphic user interface in Java to interactively change parameters of the Canny-edge filter and CHT to achieve successful detection of cells in transmitted light images. The user guide provides an example of this process to help with intuitive selection of parameter values. The user is prompted to adjust the scale of the image such that the cell size is similar to the example provided in the user guide. This attempts to ensure that the default radius range used during CHT voting process works well. Similarly, edge detection and additional CHT parameters can be chosen by comparison to the example images of these stages. The centroid positions are transformed back to the original scale at the end of the detection step, before proceeding with tracking cells.

### 2.2 Tracking

Tracking in TIAM is carried out in two steps. In the first step, a modified nearest neighbor association algorithm is applied to the outputs of the cell detection step to yield short track ‘segments’ (Fig. S3a). At each time step *t*, each cell is linked to the spatially nearest detected cell of the previous time step *t* − 1, provided the nearest detected cell is within a maximal allowed distance *r*. This process proceeds in this manner only when cells are sufficiently separated and there is no tracking ambiguity. If there is more than one cell within *r*, the algorithm returns the track segment that has been produced up to that frame and initiates new tracks with neighboring cells that caused the ambiguity. This typically happens in cases when cells cross paths or where they are present at high local density. Thus, these track segments represent sequences over which the algorithm can confidently provide tracking results. We preferred the nearest neighbor algorithm for its simplicity and intuitiveness, both in implementation and performance, when compared to the state of the art model-based tracking approaches. In addition, we prefer to use longer time-intervals to reduce phototoxicity during long-term (over an hour) multi-channel time-lapse imaging. With T cells being highly motile, longer time-intervals may not provide overlapping cells in subsequent frames, which is a restrictive requirement of contour evolution based techniques (Padfield et al., 2011). Although the nearest neighbor algorithm fails to perform well at high cell densities, as discussed later, we have obtained accurate tracking with about fifty cells in the field of view.

In the second step, an assignment algorithm is used to join shorter segments end-to-end into longer cell tracks (Fig. S3b). In order to perform segment joining, a similarity is first defined between every pair of segments based on compatibility factors such as their start/end frame, location, and speed. Then the Hungarian algorithm (Munkres, 1957) is used to find a globally optimal mapping between segments based on the similarity matrix (Bise et al., 2011; Jaqaman et al., 2008; Perera et al., 2006). Out of these mapped assignments, segments are only joined if their similarity falls above some threshold. The two-tiered approach to tracking aims to be computationally efficient by implementing an unsophisticated, greedy nearest neighbor algorithm when the tracking scenario is simple, and a more complex set of computations using the nearest neighbor results when the tracking scenario is ambiguous.

The tracking algorithms are explained in detail in the supplementary methods section along with the parameter values used. The parameters for the tracking algorithms are hard-coded in TIAM. But we have provided information in the user guide as to where in the code the parameter values can be changed if desired. Information specific to the image series can be specified through the graphic user interface in order to calculate the motility characteristics of cells (see user guide).

### 2.3 Feature extraction and data integration

TIAM is designed to make use of the multi-channel image series in order to extract additional information on tracked cells to facilitate integrative analysis and provide insights into T cell motility. The feature extraction algorithms implemented in TIAM aim to retrieve physical features such as the area of attachment to some underlying substrate (from the reflection channel), polarity (from the transmitted light channel), and fluorescence intensity (from up to two fluorescence channels), and store/report them along with motility characteristics such as the cell's speed, turn angle, arrest coefficient, and confinement index (see Supplementary methods for description). The user interface provides options to specify the channels and the features to be extracted (see TIAM user guide). Due to the consistent perspective for all image channels, tracking results from the transmitted light image channel can be directly associated with secondary channels. The centroid of cells inferred at the detection step is used to link local pixel information from these secondary channels to the tracks (Fig. 1).

Discerning the boundary contour of a given cell is a common routine that is applied to any of the image channels, which can be defined as the Region of Interest (ROI) to calculate the desired features from that image channel. Given a centroid position, a square box of a pre-determined size around the centroid is used to isolate and select the local image. This local image ideally contains only the cell of interest. For the reflection and fluorescence channels, the local image is segmented via Otsu's method (Otsu, 1979) to give the cell boundary in that channel. In order to discard pixels associated with portions of touching neighboring cells, the Watershed algorithm (Meyer, 1994) is used on the distance transform of the initial segmented image. For the transmitted light channel, Canny edge detection (Canny, 1986) is used first to discern cell boundaries in the local image. In order to discard pixels associated with portions of touching neighboring cells, the Watershed algorithm is used on the CHT of the edge image. The largest region defined by the Watershed algorithm whose centroid is within a given distance from the center of the box is considered as the cell of interest.

The local segmentation approach was primarily implemented to handle reflection image series that tend to have spatiotemporally varying foreground and background pixel intensity values, which precludes the use of global thresholding. In addition, we found during the process of implementation that the Watershed algorithm was more reliable on the local images than the global images.

### 2.4 Additional features in TIAM

TIAM allows for batch processing of experimental datasets and can automatically distinguish the cell types based on differential fluorescent vital dye-labels (see Supplementary methods and user guide). TIAM also provides the option of having the selected image channel with the outlines of cells overlaid in a tiff image series. This can provide a visual assessment of the quality of segmentation of individual cells in that channel. A stand-alone MATLAB based user interface is provided to visualize individual or pairs of tracks in the video-mode (see user guide). This allows for manual inspection of tracking results from TIAM. This user interface is also intended to help in manually recording the track and frame numbers of desired corrections in track assignments. TIAM also provides a stand-alone track-editing feature that uses the manually compiled lists of desired corrections in track assignments (see user guide). The track-editing algorithm is a two-step process, where tracks are first split at specified frames (Fig. S4). Then the specified tracks and/or sub-tracks, either resulting from breakages in the first step or the ones that were missed by the algorithm, are joined together. Icy, an open source image analysis platform, also provides a plug-in for viewing and editing tracks (de Chaumont et al., 2012).

### 2.5 Evaluation of performance of detection and tracking

Performance evaluation, also referred as performance analysis, in image analysis compares the results obtained from an automated procedure against the manually established ‘ground truth’. Herein, a ground truth track represents the ‘true’ positions of a cell as a sequence of bounding boxes. We used the Video Performance Evaluation Resource (ViPER) software (Doermann and Mihalcik, 2000) to manually draw bounding boxes around cells in each video frame and index the sequences of bounding boxes corresponding to each individual cell to designate tracks.

Performance evaluation metrics were employed to quantitatively and comprehensively assess the detection and tracking performance of TIAM and the third-party tools. We used the Sequence Frame Detection Accuracy (SFDA) and Average Tracking Accuracy (ATA) metrics (Kasturi et al., 2009) as these can be computed in a fully automated fashion and thus allow for reproducible quantification of the success of detection and tracking of objects. Further, they do not suffer from the risk of human error or bias. These metrics have been adopted as standardized metrics by the Video Analysis and Content Extraction (VACE) program (http://marathon.csee.usf.edu/vace-links.html) and the Classification of Events, Activities, and Relationships (CLEAR) consortium (www.clear-evaluation.org); which are two large-scale and community-wide efforts concerned with video tracking and interaction analysis. The metrics are based on Jaccard Similarity (Fig. S5 for intuitive illustration and and Supplementary methods for mathematical description). In order to compute SFDA and ATA, a one-to-one correspondence between ground truth and result must be established. To establish this mapping we employed the Hungarian algorithm (Munkres, 1957) with metrics based on Jaccard Similarity used to construct the similarity matrix (see Supplementary methods for details).

We have consolidated the software routines to carry out performance analysis in a separate MATLAB-based suite that we call PACT (Performance Analysis of Cell Tracking). The PACT code, its user guide and relevant ground truth datasets are available at https://github.com/willieneis/TIAM/tree/master/PACT/. The user guide also includes specific instructions on using ViPER for ground truth annotation.

### 2.6 Evaluation of performance of feature extraction

Performance of feature extraction was also evaluated against ground truth. Outlines drawn manually or by semi-automated procedures in ImageJ (Schneider et al., 2012) were listed as ROIs and used as ground truth (see Supplementary methods for details). A one-to-one correspondence between individual cells in ground truth and TIAM result was obtained using the Hungarian algorithm (Munkres, 1957). The similarity matrix for the Hungarian algorithm was constructed for each frame using the distance between centroids of every possible pairing of cells in TIAM result with those in the ground truth. Once the one-to-one correspondence is achieved, the quantified features obtained from ground truth were compared against those from TIAM.

## 3 Experimental methods

CD8 T cells were isolated from human peripheral blood mononuclear cells (from New York Blood Center) by the RosetteSep Method (StemCell Technologies). CD45RA+ve and CD45RO+ve subsets were isolated using paramagnetic beads coated with CD45RO antibody (Miltenyi Biotec). These subsets were differentially labeled with CMRA and CMFDA vital dyes (Molecular Probes) after three washes in PBS to remove trace levels of extracellular protein. Cells were cultured in phenol-red free RPMI medium supplemented with 25 mM HEPES, 1 mM sodium pyruvate and 10% fetal bovine serum (also used as imaging medium) until imaging. Fab fragments generated from TS2/4 non-blocking antibody (Huang and Springer, 1995) were labeled with Alexa Fluor 488 (Molecular Probes) and used to stain for integrin αLβ2 (LFA1) during antigen-induced motility. Pre-treatment with the TS2/4 Fab or pharmacological inhibitors was for 20 min at 37 °C. The following pharmacological inhibitors were used: myristoylated pseudosubstrate peptides of PKCα and PKCθ (20 μM; from Calbiochem) inhibit the respective kinases by binding to the active site in a competitive manner (Eichholtz et al., 1993); C20 (1 μM) is a lead compound from Boehringer Ingelheim that acts as a potent inhibitor of PKCθ by non-competitive binding to the active site (Cywin et al., 2007).

Chemokinesis experiments were performed essentially as previously described (Woolf et al., 2007). Circular coverslips were spotted sequentially with 10 μg/ml human CCL21 (R&D systems, Minneapolis, MN) for 2 h and then with 2 μg/ml murine ICAM1 for 1 h (ectodomain of ICAM1 tagged with 12× His and produced in S2 insect cells in house) at 37 °C. Majority of CD45RA+ve T cells did not show any motility on ICAM1-coated glass alone. FCS2 Bioptechs flow chambers were assembled and blocked with 5% HSA. One million cells were introduced into the flow cell and immediately imaged. Imaging was conducted at 37 °C on a Zeiss LSM710 confocal microscope operating under standard settings enclosed in an environmental chamber using a 25 × 0.8 NA oil immersion objective (equipped with a DIC prism). Spectral array detectors were set to record fluorescence from vital-dyes. Reflected light from the 543 nm laser was recorded to provide information on contact area of attached cells based on the interference with light reflected from the closely apposed plasma membrane.

Antigen induced motility was imaged in #1 8-well Labtek chambers (Nunc). The chambers were coated with 2 μg/ml each of Okt3 antibody (eBioscience) and ICAM1 for 3 h at 37 °C. Three hundred thousand cells were introduced into the wells and imaged at 37 °C on a Zeiss LSM510 confocal microscope operating under standard settings enclosed in an environmental chamber using a 40 × 1.3 NA oil immersion objective (equipped with a DIC prism). Reflection and fluorescence channels were included as described above.

## 4 Results

We evaluated the results from TIAM against manually established ground truth by visual inspection as well as by the use of quantitative metrics. We have also compared the performance of TIAM with other tools. We chose two benchmark datasets on fluorescent-labeled T cells subjected to antigen-induced and chemokine-induced motility that provided different experimental and acquisition settings as well as different motility characteristics (Table 1). We collected both DIC and fluorescence images in parallel, in order to perform tracking using both image series and compare the results.

### 4.1 Evaluation of performance of detection and tracking

Tracking of cells in transmitted light image series in TIAM is performed by a two-tiered approach that involves linkage of neighboring cells in consecutive frames followed by joining of short segments by a global optimization routine (Fig. S3). To validate the segment joining algorithm in a principled manner, we computed the ATA before and after running the algorithm on a set of ground truth tracks that had been synthetically broken. The accuracy improved drastically after joining the broken segments, which implies correct pairs of segments were joined by the algorithm (Fig. S6). Including the segment-joining algorithm in TIAM improved the ATA values for both the benchmark experiments (Fig. S7). The improvement in ATA, expectedly, was more when less than optimal *r*-value was used for the nearest neighbor association.

**Fig. 3:**
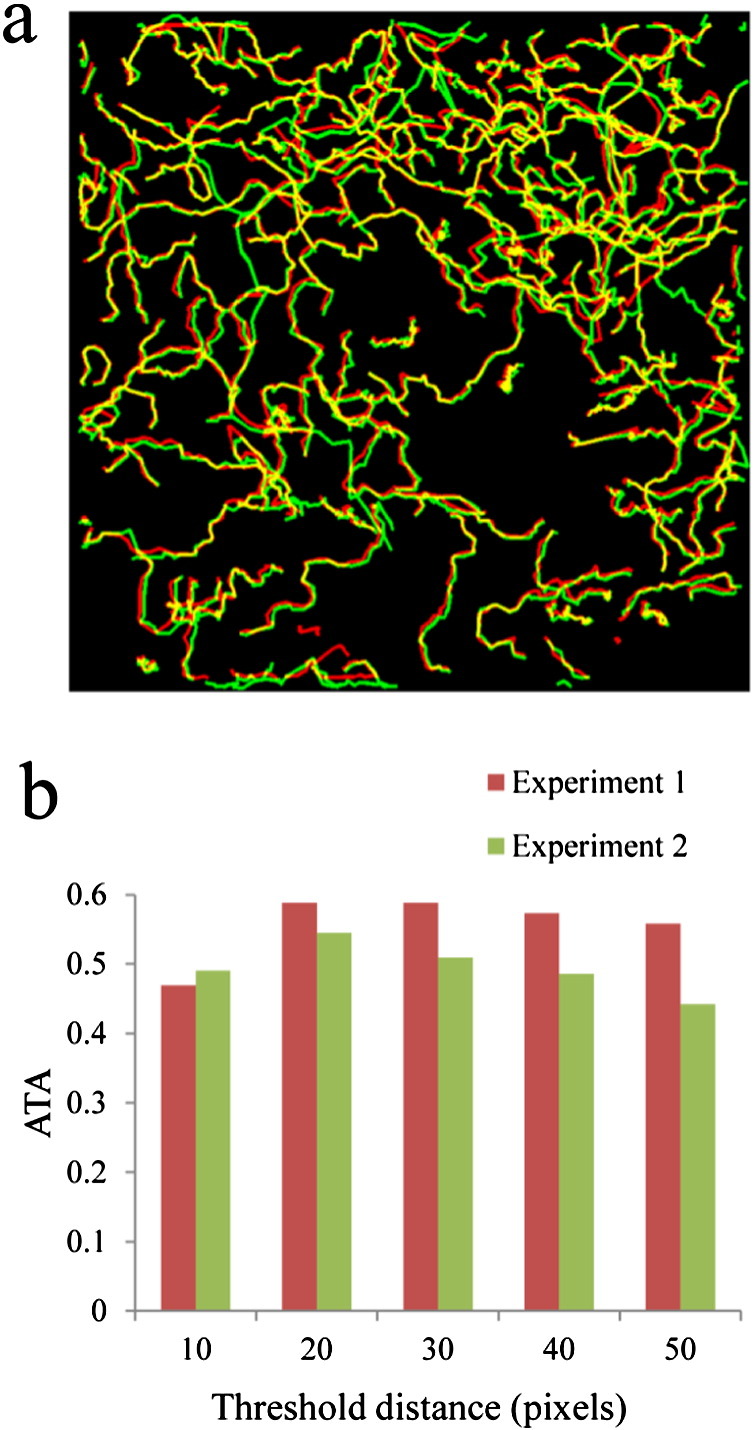
Evaluation of performance of tracking T cells by TIAM. a) Tracks of cells obtained after manually establishing the ground truth (in green) are overlaid on tracks of cells obtained from TIAM (in red). The overlap between the tracks is shown in yellow. These correspond to frames 11–40 of Experiment 2 (Table 1). b) ATA values at different thresholds for the nearest neighbor association (parameter *r*) in both experiments. ATA values suggest that tracking results are relatively robust to changes in the threshold value for the nearest neighbor association, a critical parameter in the tracking algorithm. Thresholded ATA values are plotted here. Jaccard Similarity of 0.4 or more is considered as 1 (see Supplementary methods) during the calculation of thresholded ATA. This is done to ensure that minor localization inaccuracy is not penalized.

Tracks of cells obtained from TIAM showed good overlap with those from manually established ‘ground truth’ (Fig. 3a, Videos S1 and S2). This suggests that detection and tracking results from TIAM are reliable. Visual inspection of videos revealed that the fastest moving cells escaped being tracked. In some other cases cells were not tracked continuously, leading to shorter tracks and/or multiple shorter segments (sub-tracks) corresponding to the same cell. This is most likely due to the failure of the nearest neighbor linkage during the periods of fast motility, especially in crowded areas. This observation provides an explanation for obtaining more tracks than in the ground truth and for under-estimation of mean track-length (Table 1, see below). While the modified nearest neighbor algorithm attempts to minimize the wrong track assignment by not doing any track assignment in case of ambiguity, tracking errors can nonetheless occur. In order to further characterize tracking errors, we manually recorded different types of errors in the track assignment by visual inspection using the stand-alone track visualization module of TIAM. Overall, the error rate in track assignment was estimated to be around 1% (Fig. S8). Thus, TIAM provides reliable detection and tracking of cells in transmitted light image series. Association with the nearest cell in the subsequent frame is the basis for tracking by the modified nearest neighbor algorithm in TIAM. This association is carried out if the cell in the subsequent frame happens to be within a threshold distance *r*. However, erroneous associations may occur depending on the value of this threshold distance, especially at high densities of cells or in crowded regions. In the case of TIAM, tracking accuracy is quite robust to changes in the value of threshold distance *r*, at least at the density of cells present in the benchmark experiments (Fig. 3b, Table 1).

Finally, we compared the overall performance of TIAM with some of the other well-known tools such as DYNAMIK (Jaeger et al., 2009), Icy (de Chaumont et al., 2012), Imaris (from Bitplane), and Volocity (from PerkinElmer). SFDA and ATA provide a direct way for such comparisons as they offer a single, comprehensive measure of accuracy of detection and tracking, respectively. SFDA and ATA were computed for results from all the tools on both the benchmark experiments. TIAM performed better than the other tools both in detection and tracking (Table 1, Videos S1 and S2).

### 4.2 Performance analysis of feature extraction

**Fig. 4:**
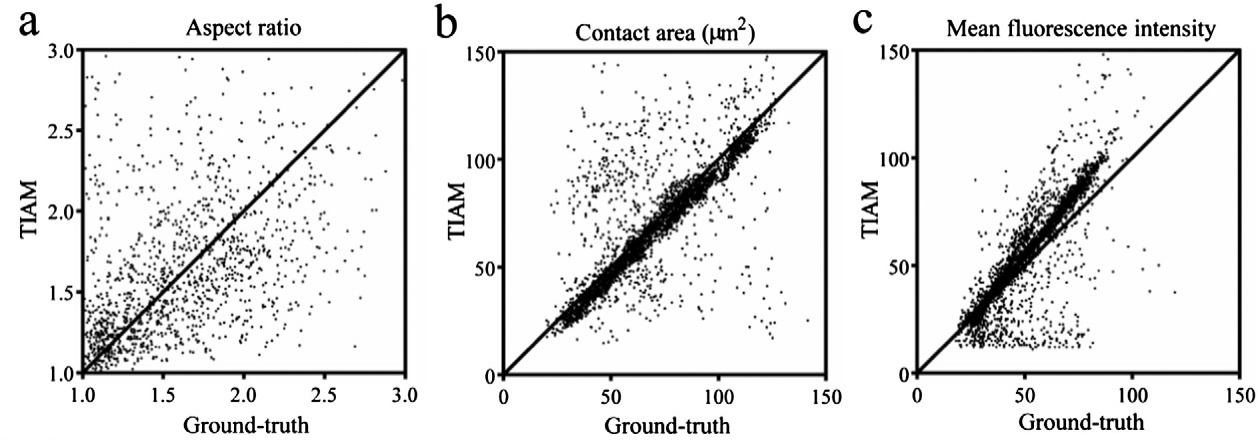
Evaluation of performance of extracting features from DIC (a), reflection (b) and fluorescence images (c). Aspect ratio (readout of morphological polarity), contact area, and mean fluorescence intensity were measured from DIC, reflection and fluorescence channels, respectively. Outlines were drawn along cell-boundaries in either a manual or semi-automated manner using ImageJ to establish the ground truth for respective channels. Performance of extracting features was evaluated by quantitative comparisons with the ground truth after establishing one-to-one pairing between TIAM results and the respective ground truth. The measured values for each pair are plotted: 1389 for DIC, 4005 for reflection and 5973 for fluorescence. Overall, the data hovered around the diagonal line implying reasonable accuracy for measurement of polarity from DIC and good accuracy for measurement of contact area and fluorescence intensity from reflection and fluorescence channels, respectively.

Extraction of features from the multi-channel image series and integration of these features with tracking results is a unique capability of TIAM. Whereas tools such as Volocity, CellProfiler and TACTICS can report on additional channels based on the mask created by global thresholding of the primary channel, TIAM handles every channel separately and performs local segmentation in each one of them. We sought to assess how well TIAM is able to perform in segmenting transmitted light, reflection, and fluorescence images and in extracting information on polarity, contact area, and mean fluorescence intensity, respectively. We again did this by comparing against ground truth that was established manually based on personal expertise. Outlines of cells in DIC, reflection and fluorescence images drawn by TIAM were in good agreement with those from the ground truth (Videos S3–S5). Measurement of aspect ratio as a readout of morphological polarity from outlines in DIC image series was reasonable, but not very good (Fig. 4a, Fig. S10). We have nonetheless decided to include it as part of TIAM due to its potential value for interpretation on the biology being studied. The contact area and mean pixel intensity of cells measured from outlines of cells from reflection and fluorescence images, respectively, were in good agreement with the ground truth (Fig. 4b and c). The median absolute error in measurements was below 10% for both (Fig. S10). The systematic bias towards higher values in reporting mean fluorescence intensity was due to higher threshold values chosen by the Otsu’s method used for local segmentation (Video S5). Along with the accuracy of calculations, processing time is also crucial to the end-user’s considerations. We have compiled the processing times for the ‘Experiment 1’ with the breakdown for individual steps of detection, tracking and feature extraction (Fig. S11).

### 4.3 Insights into motility of human CD8 T cells

**Fig. 5:**
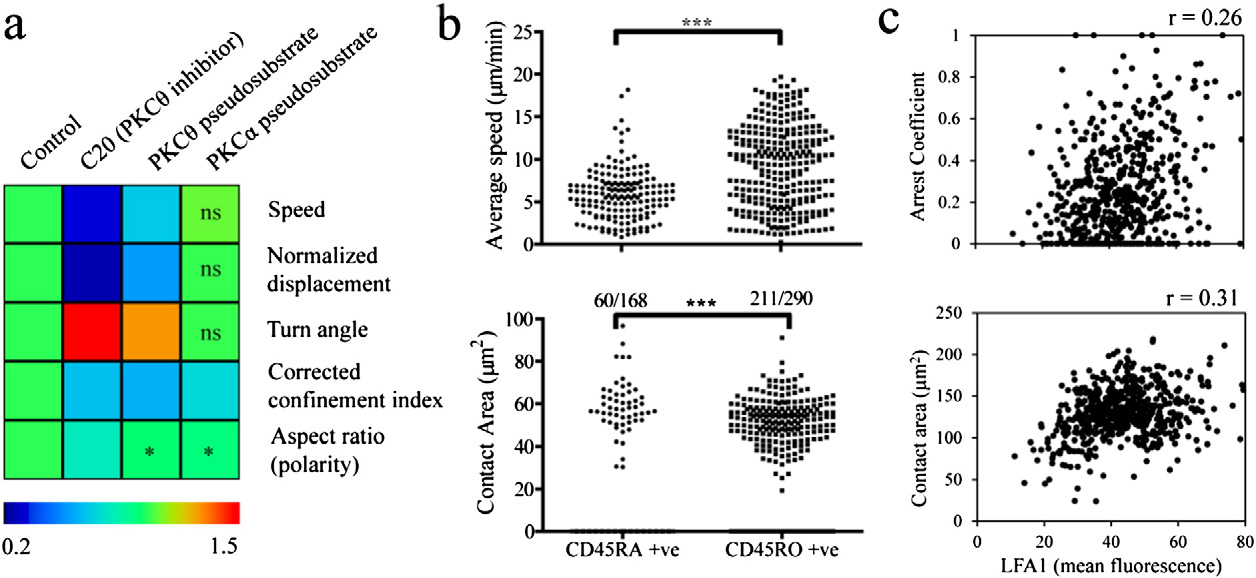
Examples of integrated analysis of human CD8 T cell motility enabled by TIAM. a) Effects of pharmacological inhibitors of PKCs on CCL21 driven chemokinesis in CD45RA+ve cells. Population median values of different motility characteristics from mean values of individual cell tracks were calculated first. These have been normalized to the median motility characteristics in the ‘control’ data and shown in a colored heat map. Statistical significance of differences in the population was calculated. Unless specified otherwise in the heat map cell, p-value was below 0.0001. b) Average speed and average contact area of CD45RA+ve and CD45RO+ve cells subjected to CCL21 driven chemokinesis is shown. The number of tracks that had reflection footprint out of the total tracked cells is given for both subsets. Even when the reflection footprint existed in a portion of the track, it was counted as a cell track with attachment. The motility experiments were conducted with a mixed population wherein the subsets were isolated, loaded with different vital dyes and then mixed in equal ratio. Statistical significance was assessed by Mann–Whitney U-test in both (a) and (b). c) Average surface density of LFA1 (measured by binding of Alexa Fluor 488 labeled Fab fragment of TS2/4 non-blocking antibody) is plotted against average contact area and arrest coefficient for individual CD45RA+ve cell tracks. Pearson correlation coefficient values are shown at the top of the plots. Arrest coefficient was calculated based on a threshold instantaneous speed of 0.5 μm/min. All results are representative of two or more independent sets of experiments.

We used TIAM to gain new insights into chemokine driven motility in primary human CD8 T cells. T cells are known to exhibit fast amoeboid motility during chemokinesis triggered by CCL21 that is coated onto a glass coverslip (Woolf et al., 2007). By using two inhibitors with different mode of action we show that PKCθ, but not PKCα, is involved in CCL21-driven chemokinesis (Fig. 5a). We also observed a concomitant decrease in morphological polarity upon inhibiting PKCθ. While the role of PKCθ is well established in T Cell Receptor (TCR) signaling, our results point to its involvement in chemokine signaling as well. The cells also exhibited an inverse relationship between speed and turn angle under the influence of inhibitors and also within the control population (Fig. 5a and Fig. S12). This is consistent with a mode of motility wherein the cells alternate between moving and turning in a motility cycle with periods of turning coinciding with a slower movement (Shenderov and Sheetz, 1997), which has also been observed in T cells (Sylwester et al., 1995). However, the observation of negative correlation within the population is novel.

We extended the use of TIAM for analyzing multi-channel image series. By differentially labeling the CD45RA and CD45RO subsets with vital fluorescent dyes, we captured the motility behavior of the two major subsets in the same experiment. By using TIAM, we were able to associate information from fluorescence and reflection images to the appropriate tracks and track-positions of cells. The CD45RO + ve cells moved faster and exhibited an increased propensity to attach to the substratum during CCL21-driven chemokinesis when compared to the CD45RA + ve cells (Fig. 5b, Video S6). Interestingly, cells from both subsets exhibited increased speed of motility when they had contact footprint in the reflection channel (Fig. S12). We also related the surface density of integrin αLβ2 (LFA1) at the immunological synapse to motility characteristics of individual cells within the CD45RA population (Fig. 5c). Surface density of LFA1 correlates with arrest coefficient and contact area of CD45RA + ve cells undergoing antigen-induced motility. These results are consistent with the crucial role played by LFA1 in promoting cell spreading and stable interactions with antigen-presenting cells (Dustin et al., 1997; Stewart et al., 1996).

## 5 Discussion

TIAM has provided multiple novel findings on the motility of T cells that were critically dependent on integrating information from DIC, reflection and two fluorescence channels. We showed that PKCθ, which was previously implicated in regulation of motility during antigen recognition (Sims et al., 2007), is also important for chemokine driven motility (Fig. 5a). We have observed that a sizeable fraction of CD45RO+ve human CD8 T cells have higher motility on CCL21- and ICAM1-coated glass compared to CD45RA+ve cells (Fig. 5b). The CD45RO subset consists of central memory and effector memory cells (Willinger et al., 2005). Central memory cells and naive cells have high expression of CCR7 whereas effector memory cells have low expression of CCR7, the chemokine receptor for CCL21. It is likely that central memory cells are the most responsive to CCL21 among all the subsets of CD8 T cells in our experiments. This is consistent with the increased speed during interstitial motility of central memory CD8 T cells compared to naive counterparts within intact lymph nodes in the absence of any antigen (Chtanova et al., 2009). Memory cells have increased surface levels of LFA1 compared to naive cells, which might contribute to higher responsiveness of central memory CD8 T cells to CCL21 co-adsorbed with ICAM1. We also observed that majority of CD45RO cells make contacts with the substratum, that are at least few microns in size, during CCL21-driven chemokinesis whereas majority of the CD45RA cells do not (Fig. 5b). These contacts are dynamic and discontinuous, similar to those observed previously in pre-activated T cells undergoing fast autonomous motility (Jacobelli et al., 2009). These contacts may also contribute to increased motility of CD45RO+ve cells.

The novel findings reported in this study were critically dependent on integrating motility information with additional information from DIC, reflection and two fluorescence channels. In the case of comparative analysis of CD45RA and CD45RO subsets, these were distinguished based on differential fluorescent dye labels. The fluorescence information allowed us to compare motility characteristics and reflection footprints of attachment simultaneously. This allowed us to delineate the motility and attachment tendencies of the subsets (Fig. 5b). Further delineation based on whether the cells within the subsets had shown contact footprint allowed us to observe that attachment promotes motility (Fig. S12). In the case of LFA1 at the contact, its surface density could be related to motility characteristics and reflection footprints of attachment (Fig. 5c).

We have brought together several existing approaches in building TIAM. The hybrid approach of edge detection followed by Hough transforms is a widely used approach for pattern recognition. Similarly the two-tier approach of linkage of neighboring objects in consecutive frames followed by temporal linkage of shorter segments is analogous to a recently introduced approach for single-particle tracking (Jaqaman et al., 2008). Put together, these approaches enable robust detection and tracking of cells. Accurate and comprehensive tracking is critical for developing motion models of cell motility and for characterizing heterogeneity in the motility behavior. Studying cellular heterogeneity has yielded better understanding of underlying mechanisms in other contexts (Altschuler and Wu, 2010). Our observation of an inverse relationship between the speed and turn angle of individual cells is a case in point (Fig. S12), as it provides evidence on the amoeboid mode of motility at the population level.

We have implemented SFDA and ATA metrics to comprehensively evaluate the performance of detection and tracking of cells on real experimental data. These metrics have gained acceptance by the computer vision research community as they facilitate standardization of procedures. Similar metrics have very recently been proposed in the cell tracking research community as well (Maska et al., 2014). As we have further demonstrated, automating the process of performance evaluation allows for comparison between multiple disparate tools, for testing the performance at different parameter settings and on different types of experimental data and for assessing the contribution of newly added features to existing algorithms. We have created a separate MATLAB-based software package that we call PACT (Performance Analysis of Cell Tracking), to enable investigators to calculate SFDA and ATA based on manually established ground truth. As individual datasets from different labs or different types of experiments are likely to be sufficiently unique, PACT can guide users to decide on the best tool to analyze their data.

Data integration is critical for extending our understanding of complex systems and processes. TIAM was structured with this overarching principle in mind to take advantage of multi-channel acquisition afforded by the state-of-the-art fluorescence microscopy platforms. TIAM is equipped to retrieve and associate features from transmitted light, fluorescence and reflection channels to cell tracks and track-positions. The insights that we obtained were critically dependent on the integrative analysis facilitated by TIAM. The generic feature extraction procedure that we have employed allows for future developments to characterize patterns in fluorescence from individual cells. It is conceivable that relating the patterns in fluorescence-based readout of critical signaling molecules to each other and to motility parameters in a spatiotemporal manner by live-cell imaging will yield rich mechanistic information (Vilela and Danuser, 2011).

## 6 Author contributions

VM conceptualized the software work-flow and oversaw the project development. WN implemented the detection and tracking algorithms and built the user interface. VM implemented the feature extraction algorithms. RM built the user interface for visualization of tracks. VM tested the software. VM conducted the experiments, established the ground truth and analyzed data. VM and WN conducted the performance analysis. VM and WN wrote the manuscript. MLD and CHW provided overall guidance. All authors discussed the results and approved the manuscript.

The following are the supplementary data related to this article.

Supplementary material.

Videos S1 and S2 Videos of benchmark experiments (1 and 2, respectively) overlaid with ground-truth track traces and results from either TIAM or Imaris (in red). The overlap is shown in yellow.

Video S3 Video of DIC image series (first 10 frames of Experiment 2) with outlines from ground-truth (in green) overlaid with outlines from TIAM (in red). The overlap is shown in yellow.

Video S4 Video of reflection image series with outlines from ground-truth (in green) overlaid with outlines from TIAM in red). The overlap is shown in yellow.

Video S5 Video of fluorescence image series (first 50 frames of Experiment 1) with outlines from ground-truth (in green) overlaid with outlines from TIAM (in red). The overlap is shown in yellow.

Video S6 Tracks of CD45RA+ve (in red) and CD45RO+ve (in green) CD8 T cells undergoing CCL21-driven chemokinesis. Reflection footprints are also included.

## Figures and Tables

**Table 1:** Comparison of performance of tracking T cells.

Qualitative characteristics	Tool name a	Number of tracks	Mean cell density (mm^− 2^)	Mean speed (μm/min)	Mean track-length (μm)	SFDA b	SFDA b ATA b (thresholded)	
Experiment 1 Antigen-induced motility of CD45RA+ve human CD8 T cells; 33.3 s between frames; 40× objective with 1.3 NA; Pixel dimensions were 0.439 μm; 225 μm square field; 100 frames	Ground-truth	125	1243.85	5.49	115.10			
	TIAM	136	1171.16	5.1	87.32	0.636	0.945	0.596
	DYNAMIK	214	715.06	5.76	44.94	0.638	0.888	0.302
	Imaris (DIC)	144	1196.84	5.87	100.64	0.412	0.678	0.508
	Icy	240	1150.62	5.19	47.8	0.574	0.882	0.380
	Imaris	147	1070.22	5.40	77.12	0.566	0.837	0.459
	Volocity	184	1212.84	5.77	80.96	0.490	0.755	0.452
Experiment 2 Chemokine-induced fast amoeboid migration of CD45RA + ve human CD8 T cells; 20 s between frames; 25× objective with 0.8 NA; Pixel dimensions were 0.664 μm; 340 μm square field; 100 frames	Ground-truth	198	923.18	9.40	158.38			
	TIAM	247	906.05	9.65	118.37	0.497	0.869	0.545
	DYNAMIK	481	497.49	11.07	37.80	0.488	0.783	0.252
	Imaris (DIC)	431	926.21	12.67	82.15	0.330	0.526	0.304
	Icy	471	989.01	9.87	66.67	0.386	0.619	0.316
	Imaris	252	946.54	9.59	127.68	0.406	0.659	0.475
	Volocity	323	988.67	9.44	102.66	0.358	0.586	0.441

a Ground-truth was established using DIC image series. Tracking on DIC image series was performed using TIAM, DYNAMIK and Imaris. Tracking was also performed on fluorescent image series of the same field collected in parallel with DIC. Icy, Imaris and Volocity were chosen for fluorescent particle tracking. Detection and tracking parameters used for each tool are described in the Supplementary methods section. Tracks shorter than 5 frames were discarded. The same ground-truth was applied to both DIC and fluorescence image series as there is excellent registration between them (Fig. S9). Similar tracking performance was observed within each tool over a range of values of maximum allowed distance for linking cells. Nonetheless, optimal values obtained for each tool is reported.

b Uniform box-sizes were considered around centroids of cells as results from Imaris and Volocity do not provide outlines of cells. Results shown here were with a box-size of 20 pixels. For thresholded performance metrics, Jaccard Similarity of 0.4 or more is considered as 1 (see Supplementary methods). Thus, minor localization inaccuracy is not penalized.
