# Supplementary material for: Integrative analysis of T cell motility from multi-channel microscopy data using TIAM
Source: J Immunol Methods. 2015 Jan;416:84–93. doi: 10.1016/j.jim.2014.11.004 (PMC4323926; doi:10.1016/j.jim.2014.11.004)
Supplement: Supplementary file 1 — Supplementary material. [file mmc1.pdf]

Integrative Analysis of T cell Motility from Multi-channel Microscopy Data using TIAM

Supplementary Material

Viveka Mayya, Willie Neiswanger, Ricardo Medina, Chris H. Wiggins, Michael L. Dustin

Content

Supplementary Methods: Detailed description of algorithms and computational methods

Subsections under Computational Methods section: Detection, Tracking (including segment joining), Data integration, Feature extraction, Motility characteristics, Visualizing and editing track assignments, Establishing the ground-truth, Cell tracking with 3rd party tools, Performance analysis, PACT.

Figure S1: Additional examples of cell detection by TIAM

Figure S2: Addtional examples of cell detection by TIAM

Figure S3: Flow chart of the tracking algorithms

Figure S4: Flow chart of the strategy for editing tracks

Figure S5: Performance evaluation of cell-tracking algorithms based on automated mapping and comprehensive metrics

Figure S6: Validation of the segment joining algorithm

Figure S7: Improvement in tracking after joining shorter segments

Figure S8: Manual assessment of errors in track assignments

Figure S9: Excellent registration between DIC and fluorescence images of T cells collected in parallel

Figure S10: Tabulation of errors in quantification of cell-associated features

Figure S11: Inverse relationship between average speed and average turn angle

Figure S12: Attachment promotes chemokinesis

Video S1 and S2: Videos of benchmark experiments (1 and 2, respectively) overlaid with ground-truth track traces and results from either TIAM or Imaris (in red). The overlap is shown in yellow.

Video S3: Video of DIC image series (first 10 frames of Experiment 2) with outlines from ground-truth (in green) overlaid with outlines from TIAM (in red). The overlap is shown in yellow.

Video S4: Video of reflection image series with outlines from ground-truth (in green) overlaid with outlines from TIAM in red). The overlap is shown in yellow.

Video S5: Video of fluorescence image series (first 50 frames of Experiment 1) with outlines from ground-truth (in green) overlaid with outlines from TIAM (in red). The overlap is shown in yellow.

Video S6: Tracks of CD45RA+ve (in red) and CD45RO+ve (in green) CD8 T cells undergoing CCL21-driven chemokinesis.Reflection footprints are also included.

Computational Methods

1. Cell Detection

TIAM employs circular Hough transform (CHT) for cell detection, which operates on a binary image of cell edges extracted from transmitted light image series. This method aims to allow for robust cell detection through instances of low contrast information, outline discontinuity and high cell density environment.

The Hough transform was developed in the 1970s as a robust method for detecting parameterized curves in images[1]. This technique converts the task of detecting complex patterns of pixels in an image (a costly global search problem) into the task of finding peaks in a parameter space. A Hough transform operates by mapping the edges of an image (found via conventional edge detection methods[2]) into a space of parameters for a given family of parametric curves for which detection is desired. This transformation produces peaks in the space at locations where many edges have "agreed" on a certain set of curve parameters. Intuitively, the Hough transform carries out a voting process, where each edge casts votes on curve parameters with which it is consistent; afterwards, the locations in the parameter space that have gained a sufficient number of votes are returned. In practice, it is often desirable only to return parameterized curves that are sufficiently different. The CHT is a special case of the Hough transform designed to locate circles parameterized by a center point, (x_0, y_0) and a radius r, and operates by searching for local maxima (that we refer to as the centroid of the cell) in the $(x_0, y_0, r) \subset \mathbb{R} \times \mathbb{R} \times \mathbb{R}_+$ parameter space. For each edge pixel, a vote is cast for each circle of a given radius r whose edge intersects that pixel.

We have used Tao Pengs implementation of the CHT (CircularHough_Grd from the MATLAB File Exchange repository) as it considers a radius range during the voting process and includes an additional parameter for searching maxima over imperfect circular shapes.

The parameters used in TIAM for detection of cells are described below:

1. resize: This is the size scaling factor for the transmitted image series. This ensures that the default radius range used during CHT voting works well for detecting cells. Through the user-interface, the user is prompted to interactively set the scale of the input images such that the cells look similar in size to those in the TIAM user-guide. The cell centroids identified based on local maxima are then scaled back to be compatible with the original image size. The default value is 1.2, as this worked well for our datasets. The graphic user-interface allows for modifying it between 0.3 and 1.7 in steps of 0.1. In the batchmode of TIAM, this parameter is called imageScale.

2. isdark: This is a binary parmater wherein a value of 1 performs a histogram equalization procedure on the transmitted light image series. This is desired if the cell boundaries are not discernible. The user-interface allows for this to be interactively set based on visual inspection. The default value is 0 as we ensured that DIC quality was typically good. In the batchmode of TIAM, this parameter is called darkImage.

3. edgevalue: This specifies the sensitivity threshold of the Canny edge filter. The default value is 0.1, as this worked well for our datasets. The graphic

user-interface allows for interactively modifying it between 0.05 and 0.5 in steps of 0.05. In the batchmode of TIAM, this parameter is called edgeValue.

4. radrange: This sets the radius range, in pixels, for the CHT voting process. This is a vector with two elements that specify the lower and upper bounds of radii with the default value set to [5,15]. The user-interface allows for interactively fine tuning this parameter if desired. But typically, if optimal size scaling factor has been chosen, the default radius range works well. In the batchmode of TIAM, this parameter is determined by radiusMin and radiusMax.

5. gradthresh: This specifies the threshold value to remove uniform intensity regions in the gradient field of the binarized image before proceeding with CHT voting. The default value is set to 10 and there is no option to change it through the user-interface. In the batchmode of TIAM, this parameter is called gradientThresh.

6. searchrad: This specifies the radius of the filter used in the search of local maxima in the hough accumulation array. Larger values of this parameter help in detecting imperfect circles. The default value is set to 15, as this typically worked well for our datasets. The graphic user-interface allows for interactively modifying it between 5 and 25 in steps of 1. In the batchmode of TIAM, this parameter is called searchRadius.

7. min_cell_sep: This specifies the minimum allowed distance between cell centroids returned by the CHT. The subsequent cell centroids in the list returned by CHT are deleted if they fall within minimum distance of previ-

ously determined cell centroids. The default value is set to 5 and there is no option to change it through the user-interface. In the batchmode of TIAM, this parameter is called minCellSeparation. Before the cell centroids are returned for tracking, those centers that have fallen outside the blobs defined by the edges are also removed.

2. Cell Tracking

Cell tracking in TIAM is carried out in two phases. The first involves a modified nearest neighbor (NN) algorithm. The NN algorithm is an unsophisticated tracking algorithm often used as a baseline procedure upon which more sophisticated methods are built. It requires as inputs the positions of the detected centroids in each frame, which are found during TIAM's cell detection procedure. At each time step t, each detected cell is associated with the spatially nearest detected cell of the previous time step $t-1$, if it is within some distance threshold. Cells without a nearest neighbor within this threshold are not given an assignment to a previously detected cell and are assumed to be a new cell. When multiple cells at a given time step are associated with the same nearest neighbor, only the spatially closest is associated, and all other cells are given no association. The nearest neighbor algorithm is given in Algorithm 1. The advantages of this algorithm include its easy implementation and straightforward behavior (many tracking algorithms that rely on more sophisticated methods have been shown to yield unexpected behavior[3]).

Algorithm 1 Nearest Neighbor Tracking Algorithm

Input:
Detected cell centroid positions for each frame in a video.
Threshold $\alpha \in \mathbb{R}_+$.
$C_{t,i}$ denotes the ith cell at time t
$C_{t,i}^{(x,y)}$ denotes the spatial coordinates of the ith cell at time t
T denotes the number of frames in a video dataset.
D_t denotes the number of detected cell positions at time t.
for $t = 2 : T$ **do**
    for $d = 1 : D_t$ **do**
        NN $\leftarrow$ arg $\min|_{i=1,\ldots,D_{t-1}} \left(\left|\left| C_{t,d}^{(x,y)} - C_{t-1,i}^{(x,y)} \right|\right| \right)$
        if $\left|\left| C_{t,d}^{(x,y)} - C_{t-1,\mathrm{NN}}^{(x,y)} \right|\right| < \alpha$ **then**
            Associate cell $C_{t,d}$ with cell $C_{t-1,NN}$.
        end if
    end for
    for $i = 1 : D_{t-1}$ **do**
        k $\leftarrow$ arg $\max|_{d=1,\ldots,D_t} \left(\left|\left| C_{t,d}^{(x,y)} - C_{t-1,i}^{(x,y)} \right|\right| \right)$
        Unassociate all cells at time t from cell $C_{t-1,i}$ except for $C_{t,k}$.
    end for
end for
Output: Sequences of associated cells (cell tracks).

The modified version of the nearest neighbor algorithm implemented by TIAM is used to form track segments. These segments are sequences of detected cell centroids over which there is a high confidence of correct tracking; the segments can be short, and do not necessarily last the entire duration that a cell is present in a video. The algorithm begins by forming the track segments and afterwards connects the segments together to form full tracks in a subsequent segment joining process. The modified nearest neighbor algorithm proceeds in the same way as the nearest neighbor algorithm, with the exception that each detected cell $C_{t,i}$ at a given frame t is only associated with a detected cell in the previous frame if

there is exactly one detected cell in the previous frame within a certain distance r. Otherwise, if there are zero or greater than one detected cells in the previous frame within a distance r from $C_{t,i}$, $C_{t,i}$ is not associated with any cell. Thus, the modified nearest neighbor algorithm chooses to forego linking than risk making a wrong tracking assignment. The modified nearest neighbor algorithm is given in Algorithm 2.

Algorithm 2 Modified Nearest Neighbor Tracking Algorithm

Input:
Detected cell centroid positions for each frame in a video.
Threshold $r \in \mathbb{R}_+$.
$C_{t,i}$ denotes the ith cell at time t
$C_{t,i}^{(x,y)}$ denotes the spatial coordinates of the ith cell at time t
T denotes the number of frames in a video dataset.
D_t denotes the number of detected cell positions at time t.
for $t = 2 : T$ **do**
  for $d = 1 : D_t$ **do**
    if $\#\left\{C_{t-1,i} \;\; s.t. \; i \in \{1, \ldots, D_{t-1}\} \; \& \; \left|\left|C_{t,d}^{(x,y)} - C_{t-1,i}^{(x,y)}\right|\right| < r\right\} \;\; == \;\; 1$ **then**
      $\text{NN} \leftarrow \arg\min|_{i=1,\ldots,D_{t-1}} \left(\left|\left|C_{t,d}^{(x,y)} - C_{t-1,i}^{(x,y)}\right|\right|\right)$
      Associate cell $C_{t,d}$ with cell $C_{t-1,NN}$.
    end if
  end for
end for
Output: Sequences of associated cells (cell tracks).

The value of r is set at 28, as it typically worked well for our datasets. But this can be changed in the code if desired (see user-guide to find the location in the code).

Segment Joining with the Hungarian Algorithm

After completion of the modified nearest neighbor algorithm, TIAM is left with a set of segments; each segment has a start-frame, an end-frame, and for all frames $t \in \{\text{start-frame}, \ldots, \text{end-frame}\}$ a set of coordinates denoting the cell centroid position at frame t. The goal of segment joining is to find segments whose start and end frames might represent disjointed subsequences of the same cell's true sequence of detected centroid positions.

In order to accomplish this task, a similarity matrix S is constructed. Letting N denote the number of subtracks produced by the modified nearest neighbor algorithm, S is of size $N \times N$. Each entry $S_{i,j}$ of this similarity matrix is a real-valued metric intended to represent the likelihood that subtrack j and subtrack i correspond to the same cell with subtrack j immediately following subtrack i. $S_{i,j}$ is defined so that incompatible segment pairings receive a very low score. $S_{i,j}$ is obtained by calculating four distinct types of (in)compatibility scores between segments as described below:

$$S_{i,j} = \begin{cases} 0 \text{ if } 1 > gap_{\mathrm{i,j}} \geq 7 \text{ or } dist_{\mathrm{i,j}} \geq 70 \\ s_{pp} + s_{ss} - s_{ds} - s_{sc} \text{ if } 1 \leq gap_{\mathrm{i,j}} < 7 \text{ and } dist_{\mathrm{i,j}} < 70 \end{cases} \tag{1}$$

where $gap_{\mathrm{i,j}}$ denotes the gap in frames between the last frame of subtrack i and first frame of subtrack j; $dist_{\mathrm{i,j}}$ denotes the euclidean pixel distance between the last position of subtrack i and the first position of subtrack j.

The positional proximity score s_{pp} between subtrack i and subtrack j is

$$s_{pp} = \begin{cases} 1/(10 \times gap_{\mathrm{i,j}}) \text{ if } dist_{\mathrm{i,j}} < 10 \\ 1/(dist_{\mathrm{i,j}} \times gap_{\mathrm{i,j}}) \text{ if } dist_{\mathrm{i,j}} \geq 10 \end{cases} \tag{2}$$

The speed similarity score s_{ss} between subtrack i and subtrack j is

$$s_{ss} = \begin{cases} 1/100 \text{ if } |\overline{v_{\mathrm{i}}} - \overline{v_{\mathrm{j}}}| \leq 1 \\ 1/(100 \times |\overline{v_{\mathrm{i}}} - \overline{v_{\mathrm{j}}}|) \text{ if } |\overline{v_{\mathrm{i}}} - \overline{v_{\mathrm{j}}}| > 1 \end{cases} \tag{3}$$

where $\overline{v_{\mathrm{i}}}$ denotes the average instantaneous speed of subtrack i in the last 5 frames; $\overline{v_{\mathrm{j}}}$ denotes the average speed of subtrack j in the first 5 frames. If any of the subtracks is shorter than 5 frames, average from the available data is used.

The directional similarity score s_{ds} between subtrack i and subtrack j is

$$s_{ds} = \begin{cases} (\theta_{\mathrm{i,ij}} \times 0.0001/gap_{\mathrm{i,j}}) + (\theta_{\mathrm{i,j}} \times 0.00005/gap_{\mathrm{i,j}}) \text{ if } gap_{\mathrm{i,j}} \leq 2 \\ (\theta_{\mathrm{i,ij}} \times 0.0002/gap_{\mathrm{i,j}}) + (\theta_{\mathrm{i,j}} \times 0.0001/gap_{\mathrm{i,j}}) \text{ if } gap_{\mathrm{i,j}} > 2 \end{cases} \tag{4}$$

where $\theta_{\mathrm{i,ij}}$ is the turn angle, in degrees, of subtrack i with the segment that would vectorially join it with subtrack j; where $\theta_{\mathrm{i,j}}$ is the turn angle, in degrees, of subtrack i with subtrack j. Turn angle is calculated in radians first based on the directionality of movement. Thus, s_{ds} penalizes for a sharper turn in the direction, especially at smaller gaps.

The speed compatibility score is s_{sc} between subtrack i and subtrack j is

$$s_{sc} = \begin{cases} 0.001 \times \Delta d_{\mathrm{i,j}} \text{ if } \Delta d_{\mathrm{i,j}} > 0 \\ 0.001 \times |\Delta d_{\mathrm{i,j}}| \text{ if } \Delta d_{\mathrm{i,j}} < 0 \text{ and } gap_{\mathrm{i,j}} = 1 \\ 0.001 \times |\Delta d_{\mathrm{i,j}}|/\theta_{\mathrm{i,j}} \text{ if } \Delta d_{\mathrm{i,j}} < 0 \text{ and } gap_{\mathrm{i,j}} > 1 \end{cases} \tag{5}$$

where $\Delta d_{\mathrm{i,j}}$ is $dist_{\mathrm{i,j}} - \left(\frac{\overline{v_{\mathrm{i}}} + \overline{v_{\mathrm{j}}}}{2} \times gap_{\mathrm{i,j}}\right)$. $\theta_{\mathrm{i,j}}$ in this case is given in radians. Thus, s_{sc} penalizes for the difference between the actual distance and the distance covered by the existing speed.

The Hungarian algorithm[4] is a polynomial-time ($O(n^3)$) solution to the problem of finding an optimal correspondence between the elements of two sets, given a matrix containing a similarity score for each pair of elements in the Cartesian product of the two sets. In this case, the similarity matrix S that we have constructed provides an optimal way to join each subtrack i to another subtrack j, where each subtrack may only be joined to one other subtrack, and may only have one other subtrack joined to it. It was desirable not to join segments that yielded smaller positive similarity score, even when they are found to be part of an optimal mapping by the Hungarian algorithm, as these subtracks likely have a low probability of representing the same cell. In order avoid spurious joinings, segments i and j are only joined if their similarity matrix value $S_{i,j}$ is greater than a threshold value δ. The value of δ is set at 0.015, as it typically worked well for our datasets. But this can be changed in the code if desired (see user-guide to find the location in the code).

4. Data Integration

Multiple image channels of the same field may be recorded using different methods of microscopy or by exciting different flurophores in the cells with specific wavelengths of light. We use transmitted light image channel (DIC, phase-contrast, or bright field) for detecting and tracking cells. Each additional image channel that is included captures additional characteristics of the cells under investigation. Extracting information from all the channels allows for integrative analysis of cell behavior. Due to the consistent perspective for all image channels, tracking results gained from the transmitted light image channel can be directly associated with secondary channels. This is done by taking the local pixel information from these secondary channels in the vicinity of the centroids of cells inferred at the detection step.

The MATLAB 'cell array' data structure is used by TIAM wherein each 'cell' holds all the information related to an individual cell-track. Each 'cell' is a two-dimensinal matrix with each row corresponding to subsequent track positions and each column corresponding to a 'property' of the cell. The property could be a feature extracted from the images or a motility characteristic calculated from track positions, both of which are described in the following two sections. Further details of the 'cell array' data structure can be found in the TIAM user guide.

5. Feature Extraction

TIAM is equipped with algorithms for extracting additional features using image data from the set of included channels. A central method here is TIAM's outline

extraction algorithm, which is explained in detail in the main text. TIAM aims to carry out feature extraction in a way that is robust to global spatiotemporal variations in pixel intensity and high cell density environments. In the current deployment, TIAM calculates polarity (see next section) from the outline/edge extracted from the transmitted-light image channel, area of attachment to the underlying substrate from the reflection (IRM) image channel and the average fluorescence value of the cell for the two fluorescence channels that TIAM accommodates. If two fluorescence channels are included in the analysis, TIAM automatically assigns a label that signifies if the cell predominantly has one type of fluorescence over the other. This is done by comparing the min/max normalized mean intensities of cells averaged over the respective tracks.

(see next page for tabulation of motility characteristics)

6. Motility Characteristics

**Parameter**	**Description**	**Comments**
Speed	Distance over time between consecutive time steps.	Descriptor for each time-step, each cell.
N-step smoothed speed	Distance over time between '$N/2$' steps before and '$N/2$' steps after the current time step.	Descriptor for each time-step, each cell.
Normalized Displacement	Distance between first and last step divided by number of steps.	Average descriptor; for each cell-track.
Corrected confinement index (same as meandering index, straightness index, and chemotactic index)	Confinement index is overall displacement divided by the track length; Corrected confinement index is confinement index multiplied by square root of the duration of the cell track[5].	Average descriptor; for each cell-track.
Arrest coefficient	Fraction of time steps for which the speed is below a threshold[5].	Average descriptor; for each cell-track.
Turn angle	Angle between direction vectors formed by the (vector) difference of cell position at consecutive steps[5]. Only computed if the centroid of the cells has moved greater than a threshold number of pixels (currently set at 0) from the previous position.	Descriptor for each time-step, each cell.
Polarity	Three readouts of polarity are given. Eccentricity and aspect ratio (major axis/minor axis) are determined by the ellipse fit to the ROI defined by the cell outline. Ellipse fitting in MATLAB is done by calculating second central moments. Circularity is the third readout and is defined as $4\pi(area/perimenter^2)$. Circularity of a circle is 1.	Descriptor for each time-step, each cell.

Speed is usually defined as the distance between centroid positions over consecutive time-steps divided by the time between steps. However, there can be issues with this definition of speed: some cell movement may be attributed to inaccuracies in the cell detection procedure, and some may be attributed to the effects of cells 'wiggling' in place. Thus, even those cells that are not actually moving, may have considerable speed attributed to them. To reduce the contribution of these factors, it is useful to compute the 'N-step smoothed speed', also referred as displacement speed, which is the distance between '$N/2$' steps before and '$N/2$' steps after the current time step divided by $N \times$ the time between steps.

7. Visualizing and editing track assignments

A stand-alone MATLAB based user-interface is provided to visualize individual or pairs of tracks in the video-mode (see user-guide). This allows for manual inspection of tracking results from TIAM. This user-interface is also intended to help in manually recording the track and frame numbers of desired corrections in track assignments. TIAM also provides a stand-alone track editing feature that uses the manually compiled lists of desired corrections in track assignments (see user-guide). The track editing algorithm is a two-step process, where tracks are first split at specified frames (Figure S4). Then the specified tracks and/or sub-tracks, either resulting from breakages in the first step or the ones that were missed by the algorithm, are joined together. In cases where there is a temporal gap between joined segments, the position of a cell is linearly extrapolated through the gap. Track associated parameters are recalculated as per new positions. Associated physical features from the last frame before the gap are copied onto the newly

created positions in the gap. It is to be noted that the track editing feature does not allow the following possibilities: modification of detection positions; creation or deletion of cells or tracks.

8. Establishing the Ground-truth

Ground-truth for performance analysis of tracking results

Ground-truth, which describes the true sequences of each cell's position and size, was fully authored by humans using the Video Performance Evaluation Resource (ViPER) tool[6]. This tool allows bounding boxes to be drawn for objects in the video and stores spatial location and dimensions of the bounding boxes. Bounding box that fully enclosed the cell outline was drawn for each frame in which a given cell was present in the video. The sequences of bounding boxes for each cell-track are indexed in ViPER allowing for direct comparison with results from different tracking tools. Ground-truth was authored for the two benchmark datasets described in Table 1. It took approximately 80 hours to author this ground-truth.

Ground-truth for performance analysis of polarity measurements

The first 5 frames of 'Experiment 1' and first 10 frames of 'Experiment 2' were considered for performance analysis of polarity measurements from the DIC channel. Outlines around DIC images of cells were drawn manually in ImageJ[7]. In order to better appreciate the boundaries of cells, magnified views were used while drawing the outlines. The outlines were stored as ROIs in ImageJ. 'Analyze Particles' routine in ImageJ was used to calculate the centroids and shape descriptors

of ROIs. It took approximately 12 hours to author this ground-truth.

Ground-truth for performance analysis of contact area measurements

A separate dataset (called 'nveMemA_irm' included with other benchmark datasets) was considered for performance analysis of contact area measurements from the reflection channel. Outlines around reflection images of cells were drawn by a semi-automated procedure in ImageJ. Reflection images were initially transformed by flat-field correction, median filtering, and bandpass FFT (Fourier Transform) filtering. This allowed the in-built 'Default' thresholding algorithm in ImageJ to automatically segment the cells with some degree of reliability. Watershed algorithm was used to break outlines of touching cells. Outlines had to be manually corrected mainly for elongated cells and touching cells. DIC was used as a guideline to draw outlines in cases where cells were giving the appearance of having merged. The outlines were stored as ROIs in ImageJ. 'Analyze Particles' routine in ImageJ was used to calculate the centroids and area of ROIs. It took approximately 15 hours to author this ground-truth due to manual corrections.

Ground-truth for performance analysis of fluorescence measurements

The first 50 frames of 'Experiment 1' were considered for performance analysis of fluorescence measurements from the fluorescence channel. Outlines around fluorescence images of cells were drawn by a semi-automated procedure in ImageJ. Cells were automatically segmented by applying the Yen thresholding algorithm on median-filtered images. Watershed algorithm was used to break outlines of touching cells. Outlines had to be manually corrected mainly for elongated cells,

touching cells and floating/drifting cells. DIC was used as a guideline to draw outlines in cases where cells were giving the appearance of having merged. The outlines were stored as ROIs in ImageJ. 'Analyze Particles' routine in ImageJ was used to calculate the centroids and mean fluorescence intensity of ROIs. It took approximately 25 hours to author this ground-truth due to manual corrections.

9. Comparison of cell tracking with 3rd Party Tools

Four third-party cell detection and tracking softwares were used to analyze the two benchmark videos (Table 1), in order to compare their performance with that of TIAM. The tools we considered are CellTrack[8], DYNAMIK[3], Icy[9], LSetCellTracker[10], Imaris (from Bitplane), TLA[11] and Volocity (from PerkinElmer). DIC image series were analyzed using CellTrack, DYNAMIK and Imaris. Fluorescence image series were analyzed using Icy, LSetCellTracker, Imaris and Volocity. They are either very popular tools (Imaris and Volocity) or present state of the art approaches (Icy and LSetCellTracker) for fluorescent particle tracking. The following sections describe how results were obtained with each of the third party tools. Different parameter values and combinations were used for tracking in order to find optimal results as much as possible. Optimal results are reported in Table 1 (main text).

TIAM

The following parameters were used for detection for Experiment 1: imageScale = 1.2; edgeValue = 0.1; radiusMin = 5; radiusMax = 15; gradientThresh = 10; searchRadius = 15; minCellSeparation = 5; darkImage = 0; maxTrackingJump

= 28.

The following parameters were used for detection for Experiment 1: imageScale = 1.6; edgeValue = 0.05; radiusMin = 5; radiusMax = 15; gradientThresh = 10; searchRadius = 14; minCellSeparation = 5; darkImage = 0; maxTrackingJump = 20.

CellTrack

To obtain cell detection and tracking results from the CellTrack software, we followed instructions found on the CellTrack instruction webpage: http://db.cse.ohio-state.edu/CellTrack/. Detection on the first frame in each video was performed using the 'Automated Cell Detection' option. Detection parameters were chosen manually based on detection results in the initial frame. No manual editing, improving, or resampling of cell boundaries was performed. Afterwards, the 'Combined Tracking' option was used to carry out cell tracking. The tracking results were exported from CellTrack as a text file. We found that CellTrack did not give meaningful results with several attempts of changing detection parameters. We therefore did not calculate SFDA and ATA values for results from CellTrack[8].

DYNAMIK

To obtain results from the DYNAMIK software, we followed instructions found on the 'Short Documentation' section of the DYNAMIK tool's webpage: http://www.picb.ac.cn/sysbio/DYNAMIK/. DYNAMIK's main routine, the CellTracking function, was applied to the images in each benchmark video and returned an output

table containing tracking results. DYNAMIK was used for tracking cells only in DIC image series. DYNAMIK uses a series of image processing steps to detect cells from DIC images[3]. DYNAMIK uses the Sobel operator for edge detection and thresholding. Tracking is achieved by an optimal matching of nearest cells in subsequent frames. The size limits for detection were set at 100 and 1100 for Experiment 1; and at 25 and 350 for Experiment 2. The maximum pixel distance for linking cells in subsequent frames was set at 28 for Experiment 1 and 20 for Experiment 2.

Icy

Blob Detector plug-in and Active Cells Tracker plug-in were used detection and tracking respectively as they were compatible with fully automated analysis. Kmean threshold method was used for initial seeding of segmentation routine with Radius minimum set at 8 pixels for Experiment 1 and 4 pixels for Experiment 2 for detection. 6 control points were used for segmentation by parametric snakes. For tracking Spational Distance Weight of 1 worked the best. Maximum distance for linkage was set at 28 for Experiment 1 and 20 for Experiment 2 for optimal results, as in other tools.

LSetCellTracker

The test datasets provided with the tool gave error messages and hence not used.

Imaris

'Spot Tracks (over time)' feature in Imaris was used to detect and track cells. Spots were detected with an 'Estimated diameter' of 8 μm for experiment 1 and 5 μm for experiment 2. Spots were selected by Imaris based on the 'quality' parameter. The threshold 'quality' value was reduced further to include many cells that were missed by the automated routine. Tracking was done using the 'Autoregression Motion' method. 'MaxGapSize' of 3 was used in all cases. 'MaxDistance' of 15 μm gave optimal results among different values attempted.

The 'Spot Tracks (over time)' feature provides centroids of cells as part of its results of the tracking analysis, but not the outlines or bounding boxes of cells. Detecting and tracking cells imaged in DIC using the 'Different spot sizes (growing regions)' feature in Imaris did not provide meaningful results. Thus we could not obtain outlines or bounding boxes for cells from Imaris.

Imaris was used for tracking cells in both DIC and fluorescence image series.

TLA

TLA has been shown to work well for tracking cells in transmitted light image series[11]. We were abel to achieve reasonable success with detection using default 'set-up' files. However only drifting cells were tracked. This is likely because they were moving with nearly constant velocity which were tracked without difficulty by the default Kalman filter approach.

Volocity

Objects (cells) were selected based on intensity and size thresholding. The threshold values were chosen based on visual inspection. A size guide of 50 and 25 μm^2 was used respectively for experiment 1 and 2 to separate touching objects. 'Shortest Path' tracking algorithm was chosen with the option to 'Automatically join broken tracks'. Maximum distance for linking cells was set at 15 μm for optimal results.

Volocity failed to detect cells in the DIC images. Volocity was used for tracking cells only in fluorescence image series.

Retrieving tracks and bounding boxes for cells

The tracking and feature extraction algorithms yield a position and outline contour of each cell at each frame. The outline contour is a set of (x, y) coordinate positions. In order to gain a bounding box for each cell, a rectangle $r = [x_{\min}, y_{\min}, x_{\max}, y_{\min}]$ is computed by taking the minimum and maximum x and y values over all the (x, y) coordinates in the set that composes the outline contour.

9. Performance Analysis of detection and tracking

Performance evaluation metrics were employed to quantitatively and comprehensively assess the detection and tracking performance of TIAM and the three third-party tools. We used the metrics developed by Kasturi et. al[12] as it also intends to provide a standardized way of evaluating performance. These have become well

established metrics in the computer vision field[13–15] and have been adopted by the Video Analysis and Content Extraction (VACE) program and the Classification of Events, Activities, and Relationships (CLEAR) consortium, two large-scale efforts concerned with video tracking and interaction analysis. The two metrics used to quantify the experimental results in this study are known as the Sequence Frame Detection Accuracy (SFDA), which is used to assess the quality of an object detection algorithm, and the Average Tracking Accuracy (ATA), which is used to assess the quality of an object tracking algorithm.

Computing performance metrics for the results from a given cell detection and tracking algorithm requires the ground-truth cell bounding box positions and sizes. We computed the SFDA and ATA for the results from each algorithm applied to the five benchmark videos. Computing the SFDA and ATA requires finding a mapping between the cell tracks from the ground-truth to those from the algorithm's output. The problem of finding this mapping is nontrivial, but has to be to solved in order to compute the performance evaluation metrics. An effective solution to this problem involves first specifying a performance metric and then choosing the mapping from ground-truth tracks to output tracks which yields the most favorable performance metric value. This process is described in detail by Kasturi et al.[12]; we followed the same method to find an optimal mapping. We implemented the Hungarian algorithm[4] as a polynomial-time ($O(n^3)$) solution to the problem of optimally mapping two sets of tracks once the similarity between any two tracks given some specified metric is established. Additionally, the method employed in[12] allows spurious and undetected cell tracks to be left unmapped, which is both desired and necessary in the case where there is a different number

of ground-truth and output tracks. Note that once a mapping from a collection of ground-truth tracks to a collection of result tracks has been established, one can determine which result tracks are false positives (the result tracks to which no ground-truth track is assigned) and which ground-truth tracks are true negatives (the ground-truth tracks that are not assigned to a result track). The numbers of tracks displaying both of these failures are factors in the performance metrics used in this study.

Once the mapping between results and ground-truth has been established, the SFDA and ATA may be computed. The following terms are used in the definitions of these two performance metrics:

- G_i denotes the spatiotemporal region occupied by the ith ground-truth object in a video, and $G_i^{(t)}$ denotes the region occupied by the ith ground-truth object in frame t.

- D_i denotes the spatiotemporal region occupied by the ith detected object in a video, and $D_i^{(t)}$ denotes the region occupied by the ith detected object in frame t.

- N_G denotes the total number of unique ground-truth objects in a video, and $N_G^{(t)}$ denotes the number of unique ground-truth objects present at frame t.

- N_D denotes the total number of unique detected objects in a video, and $N_D^{(t)}$ denotes the number of unique detected objects present at frame t.

- N_{frames} denotes the total number of frames in a video, and $N_{\text{frames}}^{(i)}$ denotes the number of frames in which an object i (which can be a ground-truth or

detected object, depending on the context) is present in a video.

- N_{mapped} denotes the number of mapped ground-truth/detect pairs in a video, and $N^{(t)}_{\text{mapped}}$ denotes the number of mapped ground-truth/detect pairs present at frame t.

The SFDA metric quantifies the performance of an object detection algorithm as a function of the number of correct detects, false positive detects, missed (true negative) detects, and spatial allignment of detects relative to the ground-truth. The SFDA is calculated by computing the Frame Detection Accuracy at frame t ($\text{FDA}^{(t)}$) for each frame in a video sequence. The FDA provides a measure of the allignment between ground-truth and detected objects in a given frame via the overlap ratio of a ground-truth/detect pair, defined to be the ratio of the intersection of ground-truth and detect regions to the union of ground-truth and detect regions. We can write the $\text{FDA}^{(t)}$ as

$$\text{FDA}^{(t)} = \frac{\text{Overlap Ratio}}{\left(\frac{N_G^{(t)}+N_D^{(t)}}{2}\right)} \tag{6}$$

where

$$\text{Overlap Ratio} = \sum_{i=1}^{N^{(t)}_{\text{mapped}}} \frac{\left|G_i^{(t)} \cap D_i^{(t)}\right|}{\left|G_i^{(t)} \cup D_i^{(t)}\right|} \tag{7}$$

The term $N^{(t)}_{\text{mapped}}$ refers to an optimal mapping between ground-truth and detects at frame t using the Jaccard similarity between the bounding boxes as the relevant metric. Given the $\text{FDA}^{(t)}$ at each frame, the SFDA can be computed; this metric

may be viewed as the average FDA over all frames of a video sequence. We define

$$\text{SFDA} = \frac{\sum_{t=1}^{N_{\text{frames}}} \text{FDA}^{(t)}}{\sum_{t=1}^{N_{\text{frames}}} \exists \left(N_G^{(t)} \vee N_D^{(t)} \right)} \tag{8}$$

where $\exists \left(N_G^{(t)} \vee N_D^{(t)} \right)$ yields a 1 if either a detected or ground-truth object is present in frame t and a 0 otherwise.

To ignore minor inconsistencies in localization, spatial overlap between the output of a method and the ground truth can be thresholded. For a given threshold value, the Thresholded Overlap Ratio is defined to be

$$\text{Thresholded Overlap Ratio}_i^{(t)} = \frac{FDA\_T_i^{(t)}}{\left| G_i^{(t)} \cup D_i^{(t)} \right|} \tag{9}$$

where

$$FDA\_T_i^{(t)} = \begin{cases} \left| G_i^{(t)} \cup D_i^{(t)} \right|, \text{ if } \frac{\left|G_i^{(t)} \cap D_i^{(t)}\right|}{\left|G_i^{(t)} \cup D_i^{(t)}\right|} \geq \text{Threshold} \\ \left| G_i^{(t)} \cap D_i^{(t)} \right|, \text{ if } \frac{\left|G_i^{(t)} \cap D_i^{(t)}\right|}{\left|G_i^{(t)} \cup D_i^{(t)}\right|} < \text{Threshold \& nonbinary thresholding} \\ 0, \text{ if } \frac{\left|G_i^{(t)} \cap D_i^{(t)}\right|}{\left|G_i^{(t)} \cup D_i^{(t)}\right|} < \text{Threshold \& binary thresholding} \end{cases} \tag{10}$$

Thus the Jaccard Similarity for a particular pair of objects (cells) is set to 1 if it is equal to or above the set threshold for non-binary thresholding. For binary thresholding, the Jaccard Similarity is set to 0 if below the threshold. We have used non-binary thresholding in our analyses, with 0.4 as the threshold Jaccard Similarity value. Thus, SFDA calculated based on thresholded spatial

overlap predominantly penalizes missed detections and false-positive detections while ignoring minor localization inaccuracies.

The ATA metric quantifies the performance of an object tracking algorithm as a function of the spatial overlap of a mapped set of sequences of detected object positions to a set of sequences of ground-truth object positions. The ATA is calculated by first computing the Sequence Track Detection Accuracy (STDA), which can be viewed as a tracking performance measure normalized in terms of the number of objects. We can write the STDA as

$$\mathrm{STDA} = \sum_{i=1}^{N_{\mathrm{mapped}}} \frac{\sum_{t=1}^{N_{\mathrm{frames}}} \left(\frac{\left| G_i^{(t)} \cap D_i^{(t)} \right|}{\left| G_i^{(t)} \cup D_i^{(t)} \right|} \right)}{N_{(G_i \cup D_i \neq \emptyset)}} \tag{11}$$

where N_{mapped} refers to an optimal mapping between ground-truth and detected objects using the numerator of the STDA as the relevant metric, and $N_{(G_i \cup D_i \neq \emptyset)}$ denotes the number of frames in which a given tracked object, the ground-truth object to which it is mapped, or both, are present. Given the STDA for a video sequence, the ATA can be computed by

$$\mathrm{ATA} = \frac{\mathrm{STDA}}{\left(\frac{N_G + N_D}{2} \right)} \tag{12}$$

ATA can also thresholded to ignore minor localization inaccuracies by thresholding the Jaccard Similarity used in the STDA formula.

10. PACT

We have consolidated the software routines to carry out performance analysis in a MATLAB-based suite that we call PACT (Performance Analysis of Cell Tracking). The PACT code, its user-guide, and relevant ground-truth data (for two benchmark experiments) are available at

https://github.com/willieneis/TIAM/tree/master/PACT/

The user-guide also includes specific instructions on using ViPER for ground-truth annotation.

PACT takes in ground-truth from ViPER as well as from a generic format described in the user-guide. Thus, manually tracked data from other tools could also be used as ground-truth. PACT takes in tracking results from TIAM as well as from the generic format. File conversion routines to convert tracking results from CellTrack, DYNAMIK, Imaris, and Volocity to the generic format are also provided. Further, PACT has been equipped to consider a default-sized box around the centroids if bounding-box information is not available.

References

1. R.O. Duda and P.E. Hart. Use of the hough transformation to detect lines and curves in pictures. *Communications of the ACM*, 15(1):11–15, 1972.

2. J. Canny. A computational approach to edge detection. *Pattern Analysis and Machine Intelligence, IEEE Transactions on*, (6):679–698, 1986.

3. S. Jaeger, Q. Song, and S.S. Chen. Dynamik: a software environment for cell dynamics,

motility, and information tracking, with an application to ras pathways. *Bioinformatics*, 25 (18):2383–2388, 2009.

4. J. Munkres. Algorithms for the assignment and transportation problems. *Journal of the Society for Industrial and Applied Mathematics*, pages 32–38, 1957.

5. J.B. Beltman, A.F.M. Marée, and R.J. de Boer. Analysing immune cell migration. *Nature Reviews Immunology*, 9(11):789–798, 2009.

6. D. Doermann and D. Mihalcik. Tools and techniques for video performance evaluation. In *Patte@articleschneider2012671, title=671 nih image to imageJ: 25 years of image analysis, author=Schneider, Caroline A and Rasband, Wayne S and Eliceiri, Kevin W and Schindelin, Johannes and Arganda-Carreras, Ignacio and Frise, Erwin and Kaynig, Verena and Longair, Mark and Pietzsch, Tobias and Preibisch, Stephan and others, journal=Nature methods, volume=9, number=7, year=2012 rn Recognition, 2000. Proceedings. 15th International Conference on*, volume 4, pages 167–170. IEEE, 2000.

7. Caroline A Schneider, Wayne S Rasband, Kevin W Eliceiri, Johannes Schindelin, Ignacio Arganda-Carreras, Erwin Frise, Verena Kaynig, Mark Longair, Tobias Pietzsch, Stephan Preibisch, et al. 671 nih image to imagej: 25 years of image analysis. *Nature methods*, 9 (7), 2012.

8. A. Sacan, H. Ferhatosmanoglu, and H. Coskun. Celltrack: an open-source software for cell tracking and motility analysis. *Bioinformatics*, 24(14):1647–1649, 2008.

9. Fabrice de Chaumont, Stéphane Dallongeville, Nicolas Chenouard, Nicolas Hervé, Sorin Pop, Thomas Provoost, Vannary Meas-Yedid, Praveen Pankajakshan, Timothée Lecomte, Yoann Le Montagner, et al. Icy: an open bioimage informatics platform for extended reproducible research. *Nature methods*, 9(7):690–696, 2012.

10. Oleh Dzyubachyk, Jeroen Essers, Wiggert A van Cappellen, Céline Baldeyron, Akiko Inagaki, Wiro J Niessen, and Erik Meijering. Automated analysis of time-lapse fluorescence microscopy images: from live cell images to intracellular foci. *Bioinformatics*, 26(19):2424–2430, 2010.

11. Johannes Huth, Malte Buchholz, Johann M Kraus, Kristian Mølhave, Cristian Gradinaru, Götz v Wichert, Thomas M Gress, Heiko Neumann, and Hans A Kestler. Timelapseanalyzer: Multi-target analysis for live-cell imaging and time-lapse microscopy. *Computer methods and programs in biomedicine*, 104(2):227–234, 2011.

12. R. Kasturi, D. Goldgof, P. Soundararajan, V. Manohar, J. Garofolo, R. Bowers, M. Boonstra, V. Korzhova, and J. Zhang. Framework for performance evaluation of face, text, and vehicle detection and tracking in video: Data, metrics, and protocol. *Pattern Analysis and Machine Intelligence, IEEE Transactions on*, 31(2):319–336, 2009.

13. A. Ellis and J. Ferryman. Pets2010 and pets2009 evaluation of results using individual ground truthed single views. In *Advanced Video and Signal Based Surveillance (AVSS), 2010 Seventh IEEE International Conference on*, pages 135–142. IEEE, 2010.

14. M. Taj, E. Maggio, and A. Cavallaro. Multi-feature graph-based object tracking. *Multimodal Technologies for Perception of Humans*, pages 190–199, 2007.

15. S. Lee and R. Nevatia. Speed performance improvement of vehicle blob tracking system. *Multimodal Technologies for Perception of Humans*, pages 197–202, 2009.

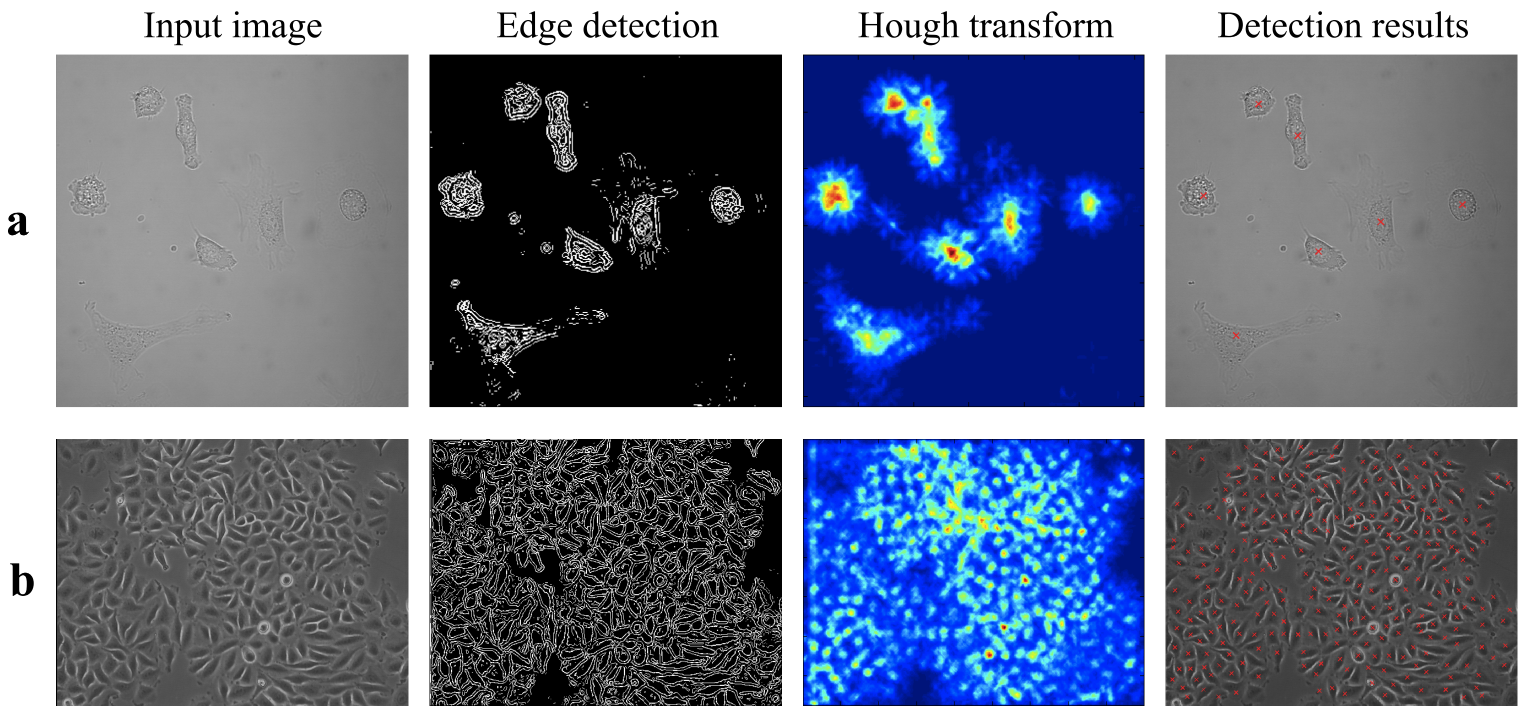

Figure S1: Additional examples of detection of cells by TIAM. Two different cell types are shown: a) NIH 3T3 fibroblasts imaged by bright field; b) SiHa cervical carcinoma cells imaged by phase contrast microscopy. The image of SiHa cells was obtained from the JCB DataViewer. The images in columns represent sequential stages during cell detection.

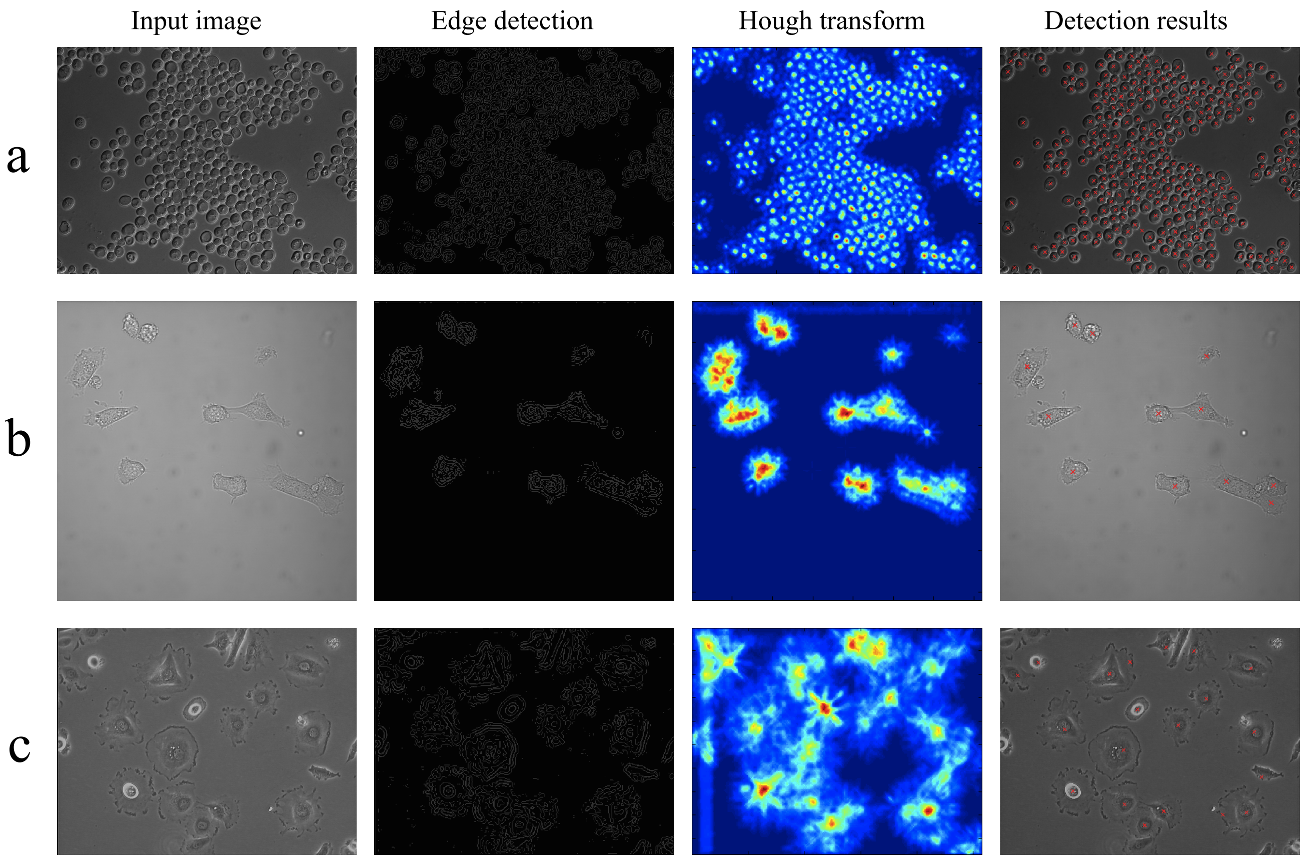

Figure S2: Additional examples of detection of cells by TIAM. Three different cell types are shown: a) yeast Saccharomyces cerevisiae imaged by DIC; b) NIH 3T3 fibroblasts imaged by bright field; c) SiHa cervical carcinoma cells imaged by phase contrast microscopy. The images of yeast and SiHa cells were obtained from the JCB DataViewer. The images in columns represent sequential stages during cell detection.

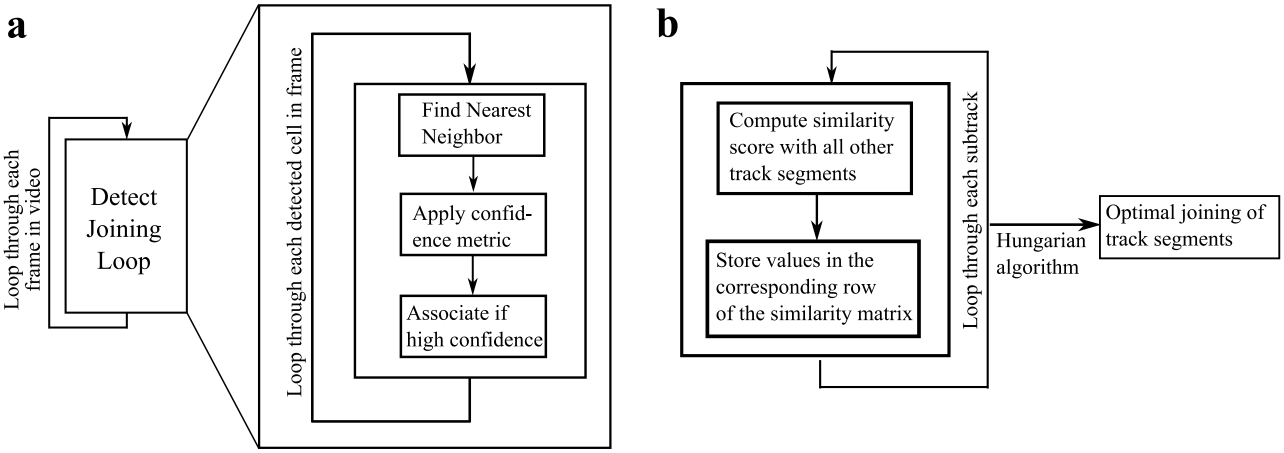

Figure S3: Flow chart of the tracking algorithms. a) Flow-chart of the modified nearest neighbor association to form track segments. Nearest neighbors within a threshold distance in the subsequent frames are considered to initiate or extend the tracks. However, a confidence metric is used to rule out ambiguous scenarios of finding multiple nearest neighbors and thus minimize track-swapping errors. This approach provides segments wherein the cells are tracked confidently. b) Flow-chart of the procedure for joining segments into longer tracks. A similarity score is first defined between every pair of segments based on factors such as their compatibility in start/end frame and spatial proximity. The Hungarian algorithm uses the similarity matrix to obtain optimal pairing between track-segments which are then joined. These are explained in detail in the Supplementary Methods section.

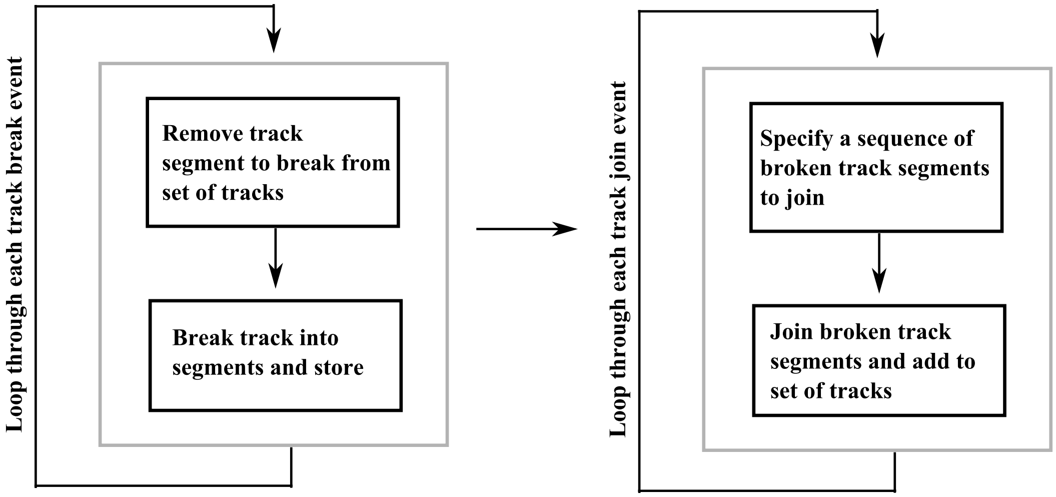

Figure S4: Flow chart of the strategy for editing tracks. The task is broken into two steps. Initially, the tracks that need to be broken are considered and are broken at specified frames. Then the specified tracks and/or sub-tracks, resulting from breakages in the first step, are joined together. This strategy can be used to correct for tracking errors.

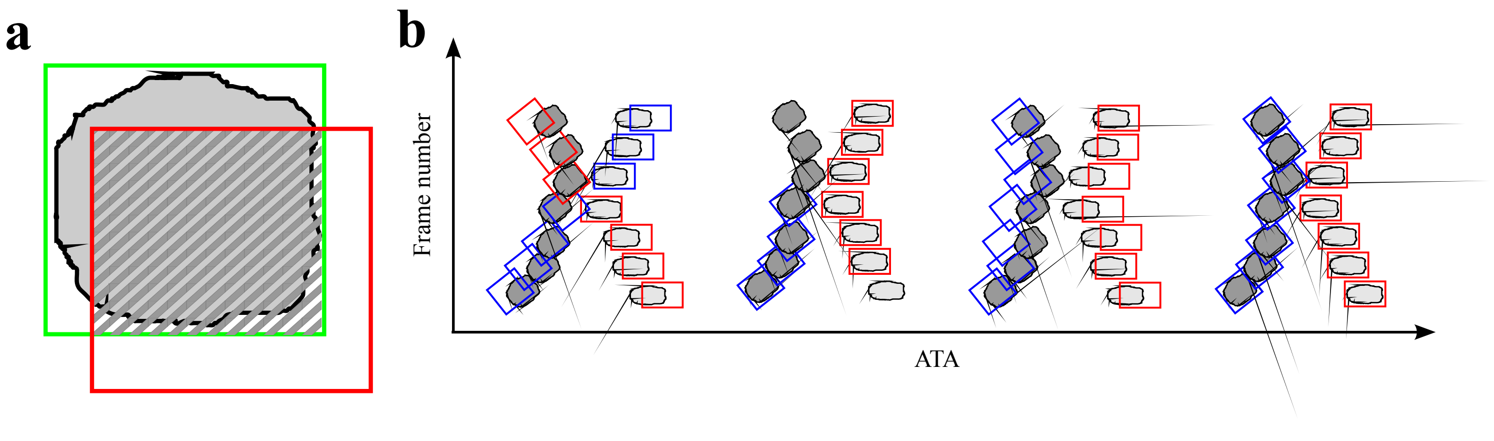

Figure S5: Performance evaluation of cell-tracking algorithms based on automated mapping and comprehensive metrics. a) Illustration to show that SFDA is based on the Jaccard similarity between the bounding boxes from ground-truth (in green) and the results of detection (in red). The intersection, denoted by shaded area, over union of the two bounding boxes is the Jaccard similarity. This is used to get optimal mapping between the objects in the ground-truth and the result by the Hungarian algorithm. SFDA is calculated on the mapped pairs by considering the Jaccard similarity over all detected objects in a frame and then over all the frames. SFDA is a measure of the accuracy of detection. b) Illustration to explain the comprehensive nature of the ATA score. Some of the common scenarios of tracking two cells by an automated procedure are arranged from left to right in the increasing order of their expected ATA value. Tracks of two cells are shown with bounding boxes in two colors. The bounding boxes represent the results of automated tracking. Jaccard similarity over the relevant frames is used to obtain an optimal mapping between the tracks in the ground-truth and the result by the Hungarian algorithm. ATA is calculated on the mapped pairs of tracks by considering the Jaccard similarity over the relevant frames and then over all the pairs of tracks. ATA is a measure of the accuracy of tracking. Both metrics have been mathematically described in the Supplementary Methods section.

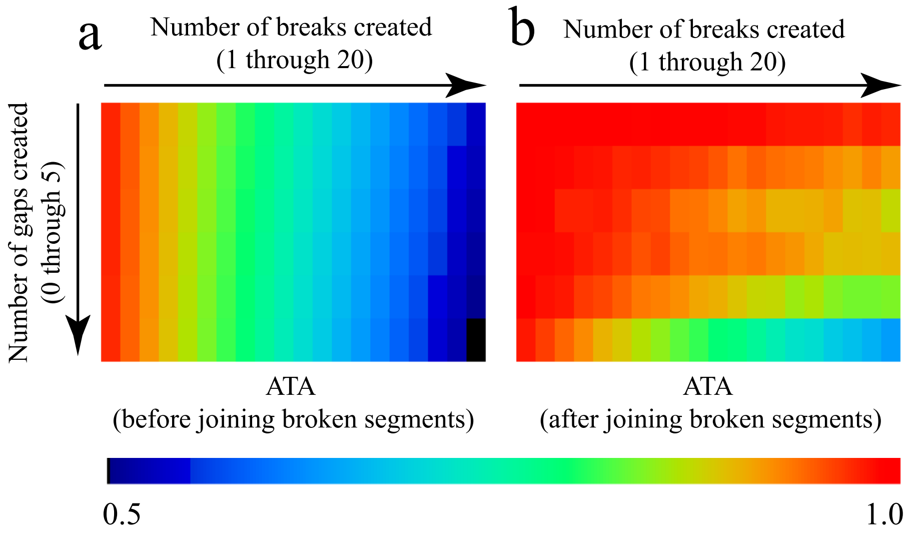

Figure S6: Validation of the segment joining algorithm. ATA values were computed before (a) and after (b) running the segment joining algorithm on a set of ground-truth tracks that had been synthetically broken. Ground-truth from the last 50 frames of 'Experiment 1' (see Table 1) was considered. Randomly chosen tracks were split at random locations with gaps ranging from 0 to 5 frames in width and number of breaks ranging from 1 to 20 in the entire set of tracks. For each pair of these two parameters, average ATA value from 50 iterations of random choices is shown in heatmap format. ATA values improved across all conditions after joining broken tracks implying that correct pair of segments were joined by the algorithm in majority of cases.

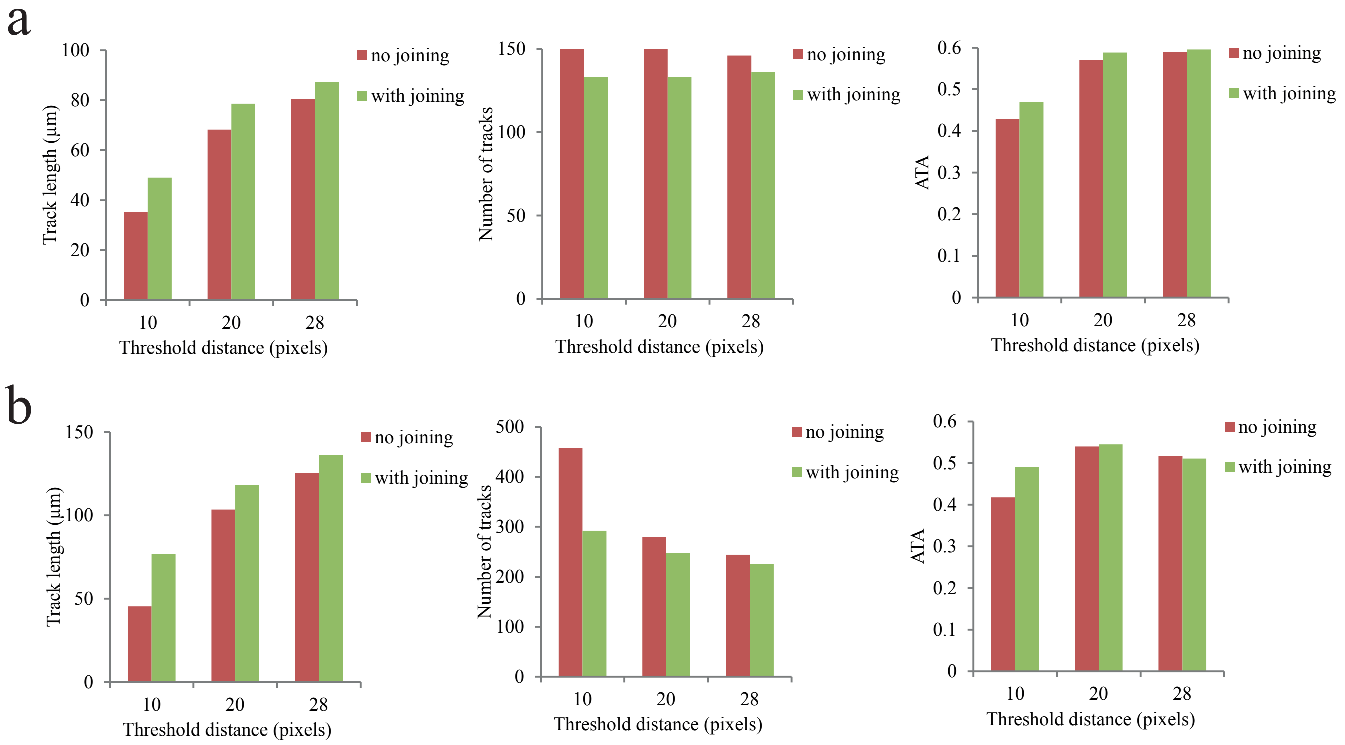

Figure S7: Improvement in tracking after joining shorter segments. (a) shows results for ‘Experiment 1’ and (b) shows results for ‘Experiment 2’. Improvement is observed in track length, total number of tracks and most importantly in ATA metric, which measures the overall tracking accuracy. The difference in the total number of tracks with and without joining shorter segments gives the number of instances of joining. There is more appreciable improvement in tracking with the implementation of the track-joining algorithm when less than optimal threshold distance (parameter *r*) is used for nearest neighbor association. Thresholded ATA values are plotted here. Jaccard Similarity of 0.4 or more is considered as 1 (see Supplementary methods) during the calculation of thresholded ATA. This is done to ensure that minor localization inaccuracy is not penalized.

Total number of track assignments	3642
Total number of tracks	89
Mutual swap-errors	16
Hybrid of swap-error and missed assignment	13
Missed assignment between full-length tracks	4
Total number of errors in track assignment	33
Percentage of incorrect and missed assignments	**0.91%** (33/3642)
ATA (results from TIAM)	0.535
ATA (after correcting errors in track assignment)	0.654

Figure S8: Manual assessment of errors in tracking. a) Tabulation of types of errors in tracking that were recorded based on manual inspection and then corrected. The last 50 frames of Experiment 1 (see Table 1) were considered. Percentage of incorrect and missed assignments, which is same as the error rate in track assignment, was found to be below 1%. The visualization module only allows for recording errors in track assignment on existing tracks. Thus the estimated error rate did not include scenarios wherein a cell was not detected/tracked over many frames. As expected, the ATA value increased after correcting the errors, thus validating the algorithm for track editing. Thresholded ATA values are considered here. Jaccard Similarity of 0.4 or more is considered as 1 (see Supplementary methods) during the calculation of thresholded ATA. This is done to ensure that minor localization inaccuracy is not penalized.

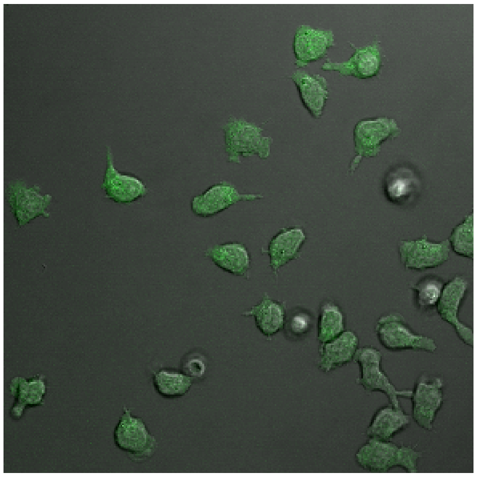

Figure S9: Excellent registration between DIC and fluorescence images of T cells collected in parallel. Image shown is from a portion of one of the frames in Experiment 1 (see Table 1). Fluorescent image series of the same field were collected in parallel with DIC. This was done in order to compare detection and tracking of cells in DIC image series by TIAM against the more widely used approaches for detection and tracking of fluorescent particles. As the registration between DIC and fluorescence images were expectedly excellent, the same ground-truth was applied to both.

	Aspect ratio from DIC image	Contact area from reflection image	Mean intensity from fluorescence image
Median absolute error	14.9%	8.0%	8.7%
Total number of cell-pairs between ground-truth and TIAM	1389	4005	5973
Number of true negatives	72 (5.2%)	14 (0.4%)	44 (0.7%)
Number of false positives	0	0	269 (4.5%)
Number of cells with value more than 20% of that of ground-truth	270 (19.4%)	312 (8%)	531 (8.9%)
Number of cells with value less than 20% of that of ground-truth	221 (15.9%)	529 (13.2%)	674 (11.3%)

Figure S10: Tabulation of errors in quantification of cell-associated features. Cells in the ground-truth were paired with cells from TIAM based on distance between them using the Hungarian algorithm. Once they were paired error in the measured cell-associated feature could be calculated. True negatives are ground-truth cells that did not have a match in TIAM results. False positives are cells in TIAM results that did not have a match in ground-truth. The true-negatives were due to missed detection and/or tracking. A high number of false-positives under fluorescence measurement were mainly floating and drifting cells that were away from the focal plane (Video S3 and Video S1). These were not outlined in the ground-truth and hence were not paired with the ground-truth. The first 5 frames of 'Experiment 1' and first 10 frames of 'Experiment 2' were considered for performance analysis of polarity measurements from the DIC channel. The first 50 frames of 'Experiment 1' were considered for performance analysis of fluorescence measurements from the fluorescence channel. A separate dataset (called 'nveMemA_irm', provided with other benchmark datasets) was considered for performance analysis of contact area measurements from the reflection channel.

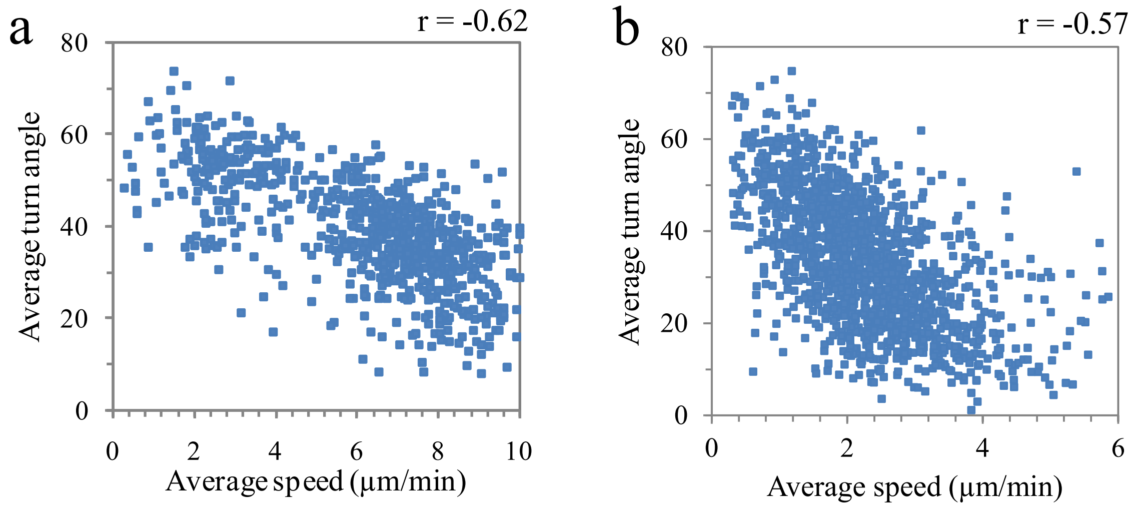

Figure S11: Inverse relationship between average speed and average turn angle of CD45RA+ve CD8 T cells undergoing ccl21 driven chemokinesis (a) or antigen-induced motility (b). Thus, the inverse relationship is observed irrespective of whether cells move with (as in antigen-induced motility) or without attachment to the substratum. Pearson correlation coefficient is given on top.

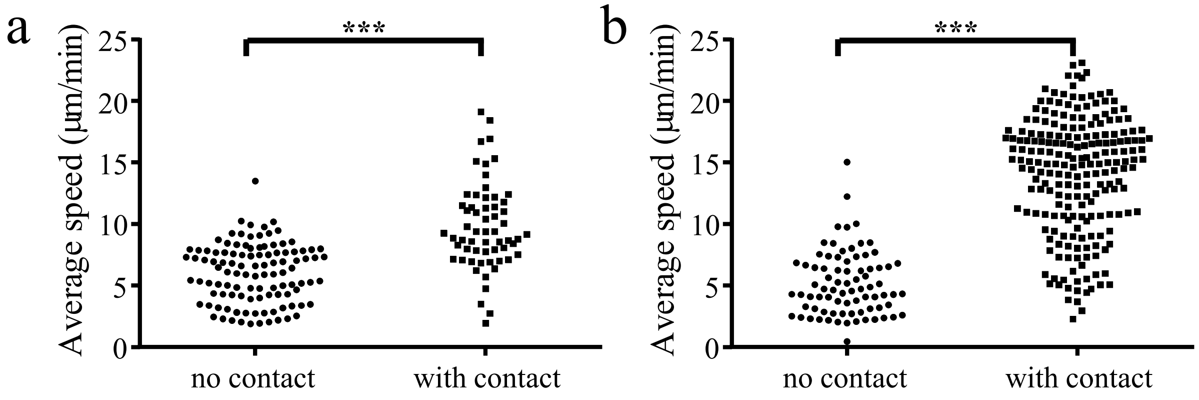

Figure S12: Attachment promotes chemokinesis. Cells that show contact footprint in the reflection channel have increased speed compared to those that do not show contact footprint, both in the CD45RA subset (a) and in the CD45RO subset (b).
