# Supplementary figures and images for: Integrative analysis of T cell motility from multi-channel microscopy data using TIAM
Source: J Immunol Methods. 2015 Jan;416:84–93. doi: 10.1016/j.jim.2014.11.004 (PMC4323926; doi:10.1016/j.jim.2014.11.004)

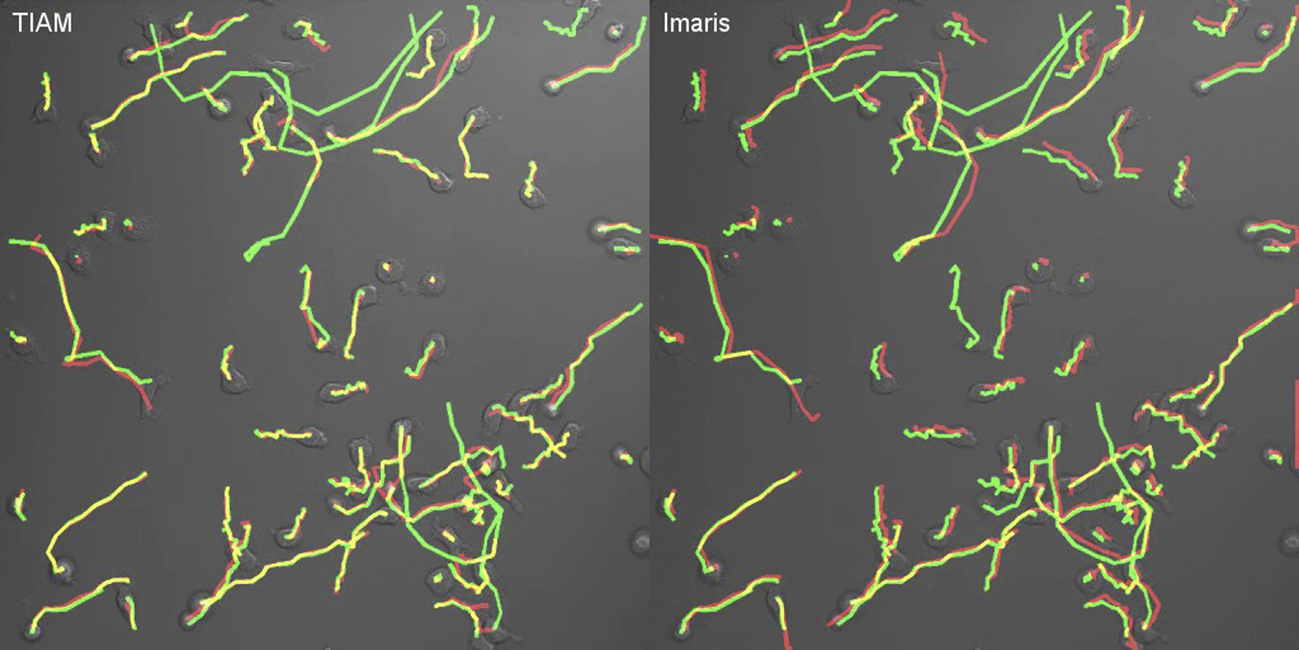

Supplement: Videos S1 and S2 — Videos of benchmark experiments (1 and 2, respectively) overlaid with ground-truth track traces and results from either TIAM or Imaris (in red). The overlap is shown in yellow. [file mmc2.jpg]

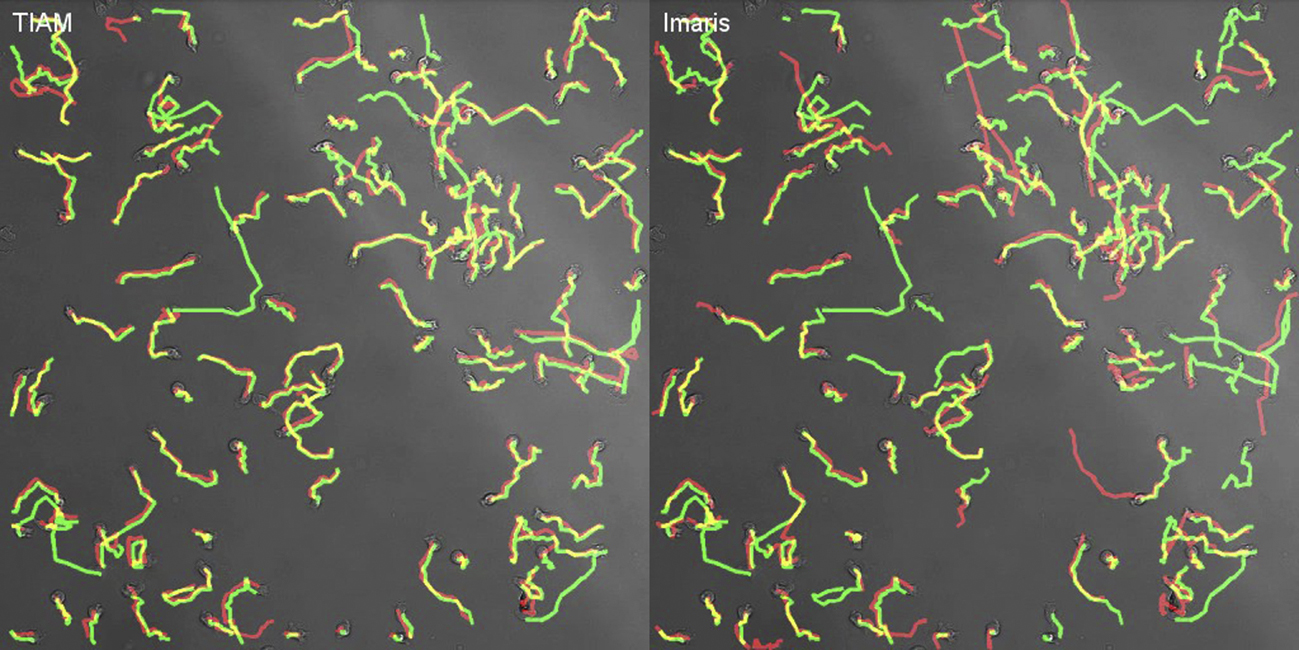

Supplement: Videos S1 and S2 — Videos of benchmark experiments (1 and 2, respectively) overlaid with ground-truth track traces and results from either TIAM or Imaris (in red). The overlap is shown in yellow. [file mmc3.jpg]

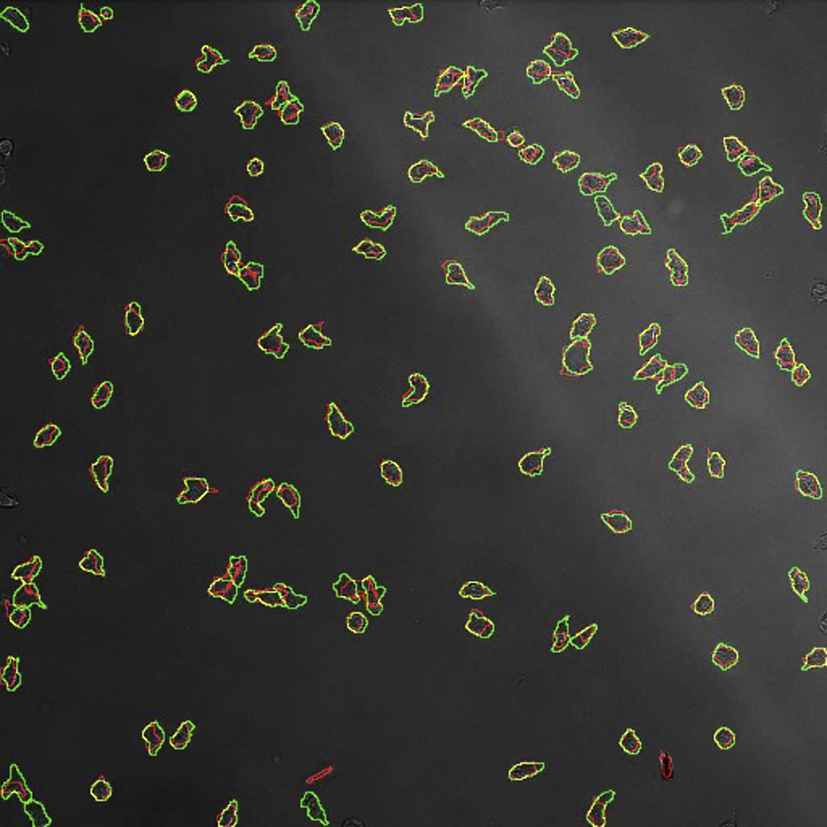

Supplement: Video S3 — Video of DIC image series (first 10 frames of Experiment 2) with outlines from ground-truth (in green) overlaid with outlines from TIAM (in red). The overlap is shown in yellow. [file mmc4.jpg]

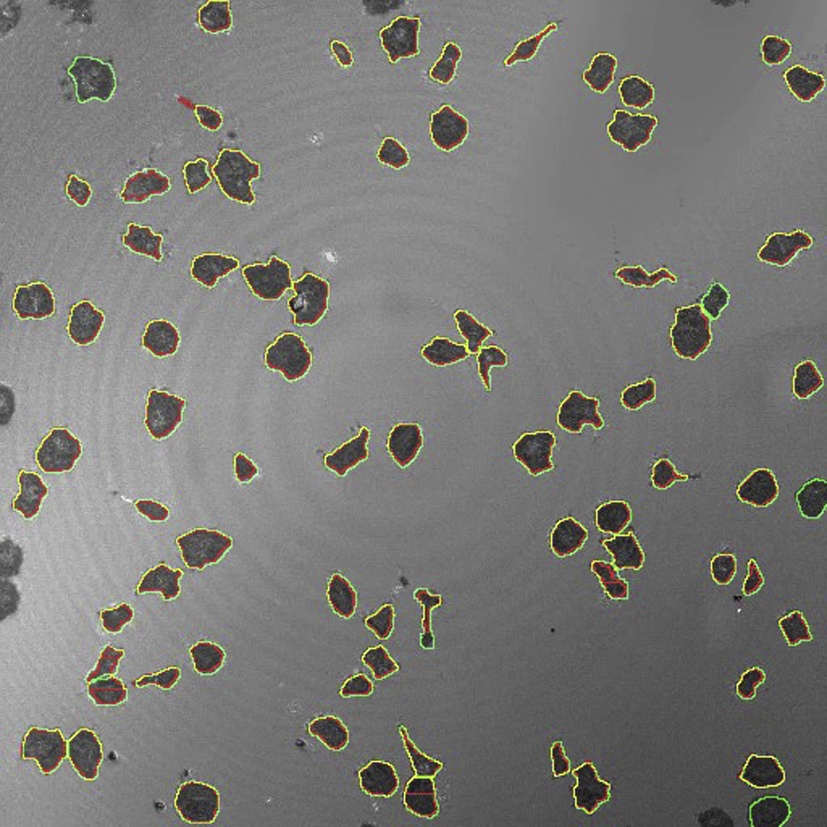

Supplement: Video S4 — Video of reflection image series with outlines from ground-truth (in green) overlaid with outlines from TIAM in red). The overlap is shown in yellow. [file mmc5.jpg]

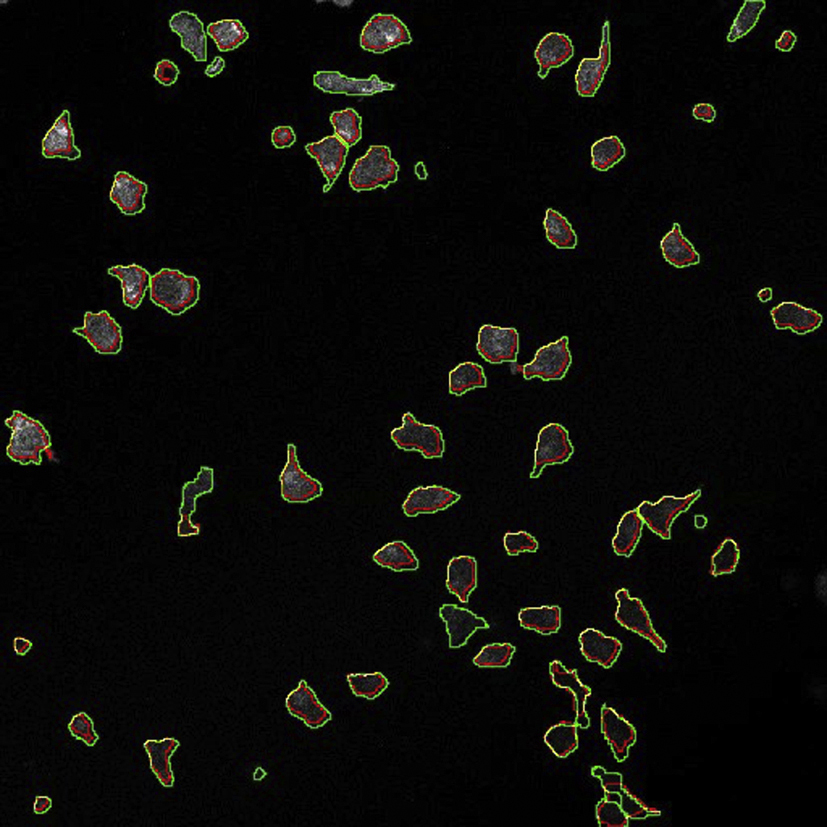

Supplement: Video S5 — Video of fluorescence image series (first 50 frames of Experiment 1) with outlines from ground-truth (in green) overlaid with outlines from TIAM (in red). The overlap is shown in yellow. [file mmc6.jpg]

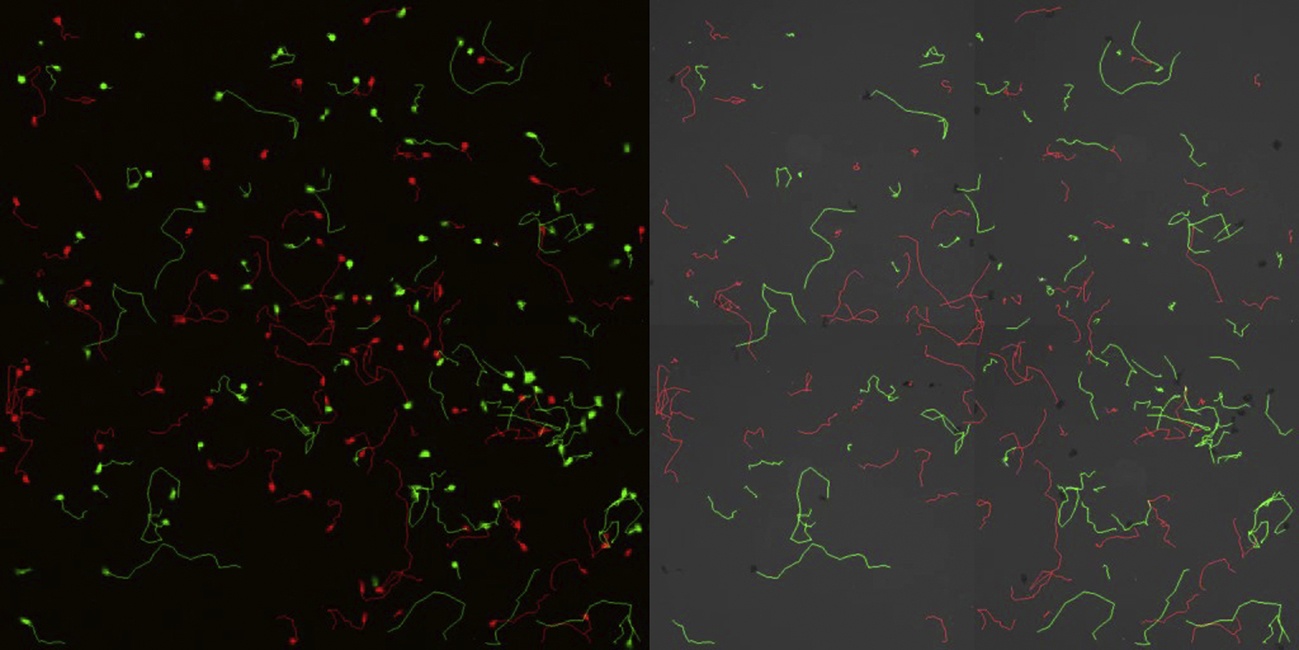

Supplement: Video S6 — Tracks of CD45RA+ve (in red) and CD45RO+ve (in green) CD8 T cells undergoing CCL21-driven chemokinesis. Reflection footprints are also included. [file mmc7.jpg]
